# Supplementary material for: Feasibility of real-time capture of routine clinical data in the electronic health record: a hospital-based, observational service-evaluation study
Source: BMJ Open. 2018 Mar 8;8(3):e019790. doi: 10.1136/bmjopen-2017-019790 (PMC5855191; doi:10.1136/bmjopen-2017-019790)
Supplement: Supplementary file 3 [file bmjopen-2017-019790supp003.pdf]

ADDITIONAL TABLE A3. Consultation times for patients seen in general cardiology clinics (A) Using the PowerForm and SmartTemplate (B) Using paper-based consultation and dictated clinic letter.

	**(A) SmartTemplate**		**(B) Dictated Clinic Letter**	
	Patient	Consultation Time (minutes: seconds)	Patient	Consultation Time (minutes: seconds)
	1	11:03	1	14:07
	2	15:03	2	18:48
	3	23:26	3	15:57
	4	14:41	4	11:33
	5	10:22	5	12:57
	6	15:21	6	13:59
	7	14:36	7	19:14
	8	11:28	8	15:27
	9	14:41	9	11:12
	10	16:53	10	10:48
	11	11:21	11	10:11
	12	12:02	12	12:38
	13	08:29	13	13:36
	14	12:28	14	13:26
	15	20:01	15	12:41
	16	14:16	16	21:13
	17	12:34	17	12:05
	18	12:35	18	16:27
	19	11:36	19	10:53
	20	19:27	20	14:09
	21	11:26	21	13:50
	22	13:34	22	17:43

**Mean consultation time**		13:58		14:13
**Standard deviation**		03:30		02:57
