# Supplementary material for: Feasibility of real-time capture of routine clinical data in the electronic health record: a hospital-based, observational service-evaluation study
Source: BMJ Open. 2018 Mar 8;8(3):e019790. doi: 10.1136/bmjopen-2017-019790 (PMC5855191; doi:10.1136/bmjopen-2017-019790)
Supplement: Supplementary file 1 [file bmjopen-2017-019790supp001.pdf]

ADDITIONAL TABLE A1. Responses to GP questionnaires

Questions (posed to 93 GPs)	Cardiac outpatient report questionnaire for GPs				
How many GPs work in your practice? (93 responses)	**0-1**	**2-4**	**5+**		
	3	34	56		
	3.2%	36.6%	60.2%		
The patient is presented with a copy of the Outpatient Report on leaving the cardiac outpatient department. How important do you think this is? (93 responses)	**Not at all important**	**Slightly important**	**Somewhat important**	**Very important**	**Extremely important**
	0	0	15	48	30
	0.0%	0.0%	16.1%	51.6%	32.3%
The Outpatient Report is transmitted electronically to the patient's EMIS file immediately after the consultation. How important do you think same-day delivery of the Outpatient Report is? (89 responses)	**Not at all important**	**Slightly important**	**Somewhat important**	**Very important**	**Extremely important**
	0	4	10	34	41
	0.0%	4.5%	11.2%	38.2%	46.1%
The SNOMED codes used in the Outpatient Report will permit the transfer of data directly into the relevant EMIS fields. How useful do you think this will be? (92 responses)	**Not at all useful**	**Slightly useful**	**Somewhat useful**	**Very useful**	**Extremely useful**
	1	2	9	37	43
	1.1%	2.2%	9.8%	40.2%	46.7%
Is the layout of the Outpatient Report easy to follow? (89 responses)	**Yes**	**No**	**Somewhat**		
	74	2	13		
	83.1%	2.2%	14.6%		
Does the content of the Outpatient Report provide adequate information for your clinical needs? (92 responses)	**Yes**	**No**	**Somewhat**		
	69	5	18		
	75.0%	5.4%	19.6%		
The Outpatient Report provides a list of investigations and medications that a patient will receive after their clinic appointment. How useful do you think these sections are for your further follow up of patients? (88 responses)	**Not at all useful**	**Slightly useful**	**Somewhat useful**	**Very useful**	**Extremely useful**
	0	0	6	35	47
	0.0%	0.0%	6.8%	39.8%	53.4%
How would you rate the utility of this new Outpatient Report compared with the conventional typed letter posted to your practice? (93 responses)	**Much less useful**	**Somewhat less useful**	**Comparable**	**Somewhat more useful**	**Much more useful**
	3	3	17	19	51
	3.2%	3.2%	18.3%	20.4%	54.8%
