# Supplementary material for: Feasibility of real-time capture of routine clinical data in the electronic health record: a hospital-based, observational service-evaluation study
Source: BMJ Open. 2018 Mar 8;8(3):e019790. doi: 10.1136/bmjopen-2017-019790 (PMC5855191; doi:10.1136/bmjopen-2017-019790)
Supplement: Supplementary file 2 [file bmjopen-2017-019790supp002.pdf]

ADDITIONAL TABLE A2. Responses to patient questionnaires

Questions (posed to 53 Patients)	Cardiac outpatient report questionnaire for patients				
Which age group are you a part of? (53 responses)	**16-35**	**36-55**	**56-75**	**76+**	**Prefer not to say**
	7	14	24	8	0
	13.2%	26.4%	45.3%	15.1%	0.0%
What is your gender? (53 responses)	**Male**	**Female**	**Prefer not to say**		
	26	27	0		
	49.1%	50.9%	0.0%		
As a patient, you are given a copy of the Outpatient Report on leaving the cardiac outpatient department. How important do you think this is? (53 responses)	**Not at all important**	**Slightly important**	**Somewhat important**	**Very important**	**Extremely important**
	1	0	3	29	20
	1.9%	0.0%	5.7%	54.7%	37.7%
Is the layout of the Outpatient Report easy to follow? (53 responses)	**Yes**	**No**	**Somewhat**		
	52	0	1		
	98.1%	0.0%	1.9%		
How would you rate this new Outpatient Report compared with the conventional typed letter posted to your home? (53 responses)	**Much less useful**	**Somewhat less useful**	**Comparable**	**Somewhat more useful**	**Much more useful**
	0	0	4	12	37
	0.0%	0.0%	7.5%	22.6%	69.8%
Do you think this Outpatient Report will help you to understand your medical condition? (53 responses)	**Yes**	**No**	**Somewhat**		
	49	1	3		
	92.5%	1.9%	5.7%		
Does the Outpatient Report help you understand the follow up you will receive after your consultation? (53 responses)	**Yes**	**No**	**Somewhat**		
	49	3	1		
	92.5%	5.7%	1.9%		
