# Supplementary material for: Feasibility of real-time capture of routine clinical data in the electronic health record: a hospital-based, observational service-evaluation study
Source: BMJ Open. 2018 Mar 8;8(3):e019790. doi: 10.1136/bmjopen-2017-019790 (PMC5855191; doi:10.1136/bmjopen-2017-019790)
Supplement: Author's manuscript [file bmjopen-2017-019790.draft_revisions.pdf]

BMJ Open

BMJ Open is committed to open peer review. As part of this commitment we make the peer review history of every article we publish publicly available.

When an article is published we post the peer reviewers' comments and the authors' responses online. We also post the versions of the paper that were used during peer review. These are the versions that the peer review comments apply to.

The versions of the paper that follow are the versions that were submitted during the peer review process. They are not the versions of record or the final published versions. They should not be cited or distributed as the published version of this manuscript.

BMJ Open is an open access journal and the full, final, typeset and author-corrected version of record of the manuscript is available on our site with no access controls, subscription charges or pay-per-view fees (http://bmjopen.bmj.com).

If you have any questions on BMJ Open's open peer review process please email info.bmjopen@bmj.com

BMJ Open

Real-time capture of routine clinical data in the hospital electronic health record: benefits for communication with patients and primary care physicians

Journal:	*BMJ Open*
Manuscript ID	bmjopen-2017-019790
Article Type:	Research
Date Submitted by the Author:	28-Sep-2017
Complete List of Authors:	Bodagh, Neil; Barts Health NHS Trust, Barts Heart Centre<br>Archbold, R; Barts Health NHS Trust, Barts Heart Centre<br>Weerackody, Roshan; Barts Health NHS Trust, Barts Heart Centre<br>Hawking, Meredith; Barts and The London School of Medicine and Dentistry, Centre for Primary Care and Public Health<br>Barnes, Michael; William Harvey Research Institute, Director of Bioinformatics<br>Lee, Aaron; William Harvey Research Institute, Centre for Advanced Cardiovascular Imaging<br>Janjuha, Surjeet; Barts Health NHS Trust, Electronic Health Record Development Manager<br>Gutteridge, Charles; Barts Health NHS Trust,<br>Robson, John; Barts and The London School of Medicine and Dentistry, Queen Mary University of London, Centre for Primary Care and Public Health<br>Timmis, Adam; Bart's Heart Centre, NIHR Cardiovascular Biomedical Research Unit; University College London, Farr Institute
**Primary Subject Heading**:	Health informatics
Secondary Subject Heading:	Cardiovascular medicine
Keywords:	Health informatics < BIOTECHNOLOGY & BIOINFORMATICS, CARDIOLOGY, Information technology < BIOTECHNOLOGY & BIOINFORMATICS

SCHOLARONE™
Manuscripts

Real-time capture of routine clinical data in the hospital electronic health record: benefits for communication with patients and primary care physicians

Neil Bodagh MB ChB[1], R. Andrew Archbold MD[1], Roshan Weerackody PhD[1], Meredith K. D. Hawking MPH[2], Michael R Barnes PhD[3], Aaron M Lee PhD[4], Surjeet Janjuha HND[5], Charles Gutteridge FRCP[6], John Robson FRCGP[2] , Adam Timmis MD[1,7]

[1] Barts Heart Centre, London, UK
[2] Clinical Effectiveness Group, Queen Mary University of London, UK
[3] Director of Bioinformatics, William Harvey Research Institute Queen Mary University of London, UK
[4] Centre for Advanced Cardiovascular Imaging, William Harvey Research Institute, Queen Mary University of London, UK
[5] EHR Development Manager, Barts Health NHS Trust, London, UK
[6] Chief Clinical Information Officer, Barts Health NHS Trust, London, UK
[7] The Farr Institute of Health Informatics Research, University College London, UK

Corresponding author: Dr Neil Bodagh, Barts Heart Centre, W Smithfield, London EC1A 7BE, Tel +447774836031, Fax +44 20 3416 5000, Email: neil.bodagh@nhs.net

KEY WORDS

General Practitioners, Health Information Exchange, Outpatients, Primary Health Care, Surveys and Questionnaires

Word count: 2991

For peer review only - http://bmjopen.bmj.com/site/about/guidelines.xhtml

ABSTRACT

Objectives. The electronic health record (EHR) is under-utilised in the hospital setting. The aim of this service evaluation study was to respond to NHS Digital's ambition for a paperless NHS by capturing routinely collected cardiac outpatient data in the EHR to populate summary patient reports and provide a resource for audit and research.

Design. A PowerForm template was developed within the Cerner EHR, for real-time entry of routine clinical data by clinicians attending a cardiac outpatient clinic. Data captured within the PowerForm automatically populated a SmartTemplate to generate a view-only report that was immediately available for the patient and for electronic transmission to the referring general practitioner (GP).

Results. During the first eight months, the PowerForm template was used in 61% (360/594) of consecutive outpatient referrals increasing from 42% to 77% during the course of the study. Structured patient reports were available for immediate sharing with the referring GP using Cerner Health Information Exchange technology while electronic transmission was successfully developed in a sub-study of 64 cases, with direct delivery by the NHS Data Transfer Service in 29 cases and NHS mail in the remainder. In feedback from 53 patients and 93 GPs, the report's immediate availability was considered more useful than waiting for postal delivery of a conventional typed letter. De-identified template data for all 360 patients were successfully captured within the Trust system, confirming availability of these routinely collected outpatient data for audit and research.

For peer review only - http://bmjopen.bmj.com/site/about/guidelines.xhtml

Conclusion. Electronic template development tailored to the requirements of a specialist outpatient clinic facilitates capture of routinely collected data within the Cerner EHR. These data can be made available for audit and research. They can also be used to enhance communication by populating structured reports for immediate delivery to patients and GPs.

STRENGTHS AND LIMITATIONS

- Routinely collected clinical data in a cardiac outpatient clinic were successfully captured in the hospital electronic health record (EHR) and made available for audit and research
- Capture of relevant data permitted population of a patient report which was immediately available for presentation to the patient and electronic transfer to the referring general practitioner
- In feedback from patients and GPs, the report's immediate availability was considered more useful than waiting for postal delivery of a conventional typed letter.
- The technology applied in capturing outpatient clinical data and electronic transmission of the patient reports is potentially transportable but this will need testing in larger groups
- It was a limitation that the GPs we surveyed were restricted to one clinical commissioning group in East London and the response rate of 44% leaves the results prone to response bias.

For peer review only - http://bmjopen.bmj.com/site/about/guidelines.xhtml

BACKGROUND

The electronic health record (EHR) is a longitudinal accumulation of electronic health information collected during routine healthcare provision[1]. It has the potential to optimise documentation of patient encounters, aid communication between healthcare professionals, improve access to medical information and form a repository of clinical data for use in audit and research[2]. The EHR, however, is severely under-utilised[3] due to a variety of factors that include concerns about disruptions to workflow and difficulties with inputting medical record data[4]. The volume of missing data ensures that few audit and research outputs are based on routinely collected data within the EHR[5, 6].

Outpatient consultations are the most frequent hospital-based clinical interactions and represent an important missed opportunity to contribute clinical data to the EHR. Encounters are usually documented in a dictated clinic letter that is either stored in the paper record or scanned into an electronic repository where it cannot readily be searched or curated. The variable structure and content of the dictated clinic letter undermines its utility for communicating clinical information with previous studies affirming that general practitioners (GPs) prefer structured correspondence about the patients they refer for specialist outpatient care[7-10]. Communication is further undermined by the inherent inefficiency of the clinic letter which may take many days after the index consultation to arrive at the address of the referring primary care physician.

In the USA, there has been a drive to increase utilisation of the EHR through the "EHR Meaningful Use Programme"[11, 12]. This has led to the development of an

For peer review only - http://bmjopen.bmj.com/site/about/guidelines.xhtml

electronic after-visit summary which aims to provide patients and their referring clinicians with relevant information and actionable instructions[13]. Studies to date have demonstrated that the after-visit summary is valued by both patients and primary care physicians[14]. As far as we are aware, the potential benefits of such a document in a cardiac outpatient setting have not been examined.

The CERNER Millennium (*Cerner Millenium, Kansas, USA*) EHR system operates in >20 hospital Trusts across the UK[15] and is widely installed globally[16]. In the present study we have used Cerner's PowerChart application to develop a SNOMED-based electronic PowerForm comprising a user-friendly interface for real-time entry of clinical data during consultation in a general cardiac outpatient clinic. The aims of this study were 1) to test the feasibility of outpatient data capture in digital format for automatic development of a structured patient report, 2) to develop methods for immediate electronic delivery of patient reports to referring primary care physicians, 3) to determine the value of patient reports for improving communication with patients and primary care physicians, and 4) to confirm the availability of outpatient data entered into the PowerForm for audit and research.

METHODS

In presenting this research we have adhered to Standards for Quality Improvement Reporting Excellence (SQUIRE) guidelines for reporting new knowledge about how to improve healthcare[17].

PowerForm. Technical build experts used the Cerner PowerChart application to develop an electronic template (PowerForm) for clinical data entry according to a

For peer review only - http://bmjopen.bmj.com/site/about/guidelines.xhtml

strict specification based on the following queries: reason for referral, presenting symptoms, risk factors for cardiovascular disease and hypertension, prior cardiac procedures, examination findings, investigations ordered, diagnosis and problems, cardiac treatment and discharge/follow-up arrangements. An adaptation of Agile methodology[18] was used in developing the PowerForm in incremental steps which were each tested and modified as necessary before being added to the software bank that contributed to the final product. Ease of use was enhanced by applying conditional logic to guide data entry into those fields relevant for a particular patient. The data captured within the PowerForm populated some of the key data fields within the EHR such as “Cardiac Procedures” and “Diagnosis and Problems” using SNOMED terms throughout while the additional cardiac-specific information incorporated within the PowerForm further enriched Cerner’s digital data repository.

SmartTemplate. This was developed using the PowerChart application to pull information from the PowerForm into a highly structured “patient report” that summarised the key clinical findings. The report included the reason for referral, risk factors, vital signs, diagnosis and problems, investigations, treatment, discharge/follow-up arrangements and action points for the referring GP. Again, an adaptation of Agile methodology[18] was used in developing the SmartTemplate which was designed to replace the conventional dictated letter in providing the referring GP and the patient with necessary information about the clinic visit. The view-only report generated within the SmartTemplate was immediately available at the end of the consultation.

Patients. The outpatient PowerForm was made available to three consultant

For peer review only - http://bmjopen.bmj.com/site/about/guidelines.xhtml

cardiologists attending a weekly general cardiology clinic in Barts Health NHS Trust. New referrals seen in the clinic from 1/6/2016 to 31/1/2017 were included. Follow-up patients were excluded. Use of the PowerForm was at the discretion of the participating consultants who had the alternative option of making a conventional paper record of the consultation and dictating a clinic letter to the referring GP. Data were entered into the PowerForm in real time and at the end of the consultation there was the opportunity to present the patient with a printed copy of the patient report.

Patient report delivery to GP. The patient report, generated within the SmartTemplate, was designed for electronic delivery to the referring GP. This function was introduced incrementally, starting with Cerner Health Information Exchange (HIE) technology to mirror the patient report across the interface between primary and secondary care. This allowed for inspection of the patient report by the GP in the patient's Egton Medical Information Systems (EMIS) file without true data export. The technology for exporting the patient reports directly into the primary care record was then developed and tested in a separate sample of 64 cardiac out-patients. The technology utilised the Data Transfer Service (DTS)[19] in 125 local practices that had been appropriately configured. Upon electronic sign-off of the report at the end of the consultation the DTS delivered it directly into the patient's EMIS file. In those local practices not yet configured for DTS transmission, the reports were delivered by NHS mail.

Extraction of cardiac outpatient data. The clinical dataset recorded within the cardiac outpatient PowerForm was extracted from Trust Data Warehouse SQL server tables using a combination of queries. These data were password protected and

For peer review only - http://bmjopen.bmj.com/site/about/guidelines.xhtml

stored within the NHS Trust system and were then anonymised. Identifying information was removed, including NHS identifiers, address details, visit dates, and the identification of medical staff and centres. Retained demographic data were limited to patient gender, age and ethnicity. These anonymised data were then subjected to the aggregated analysis presented in this work. Data manipulation was performed using the Python software language and visualised using the Plotly framework (*Plotly, Montreal, Quebec, Canada*).

GP and patient surveys. In order to obtain feedback about the utility of the patient reports, 210 GPs in the Newham Clinical Commissioning Group were surveyed, using SurveyMonkey software (*SurveyMonkey Inc., Palo Alto, California, USA*) They were provided with three samples of anonymised patient reports and paired dictated letters and invited to complete the survey by answering questions about their preferences. A paper-based survey was also conducted of 53 patients who were invited to answer questions after their consultation upon receipt of their patient report. Participation in both surveys was voluntary and all responses were anonymised. The survey questions are shown in additional tables A1 and A2. An ordinal logistic regression model was used to test whether or not a patient's gender or age group could predict a patient's thoughts on the utility of the patient report compared with the conventional dictated clinic letter. The data were analysed using SPSS for Windows v.17.0 software (*SPSS Inc., Chicago, IL, USA*).

Consultation times. These were calculated in a substudy of 44 new referrals seen by one of the participating consultants in seven consecutive clinics. The consultation times for PowerForm consultations were compared with the consultation times taken

For peer review only - http://bmjopen.bmj.com/site/about/guidelines.xhtml

for conventional paper consultations. Consultation time was defined as the time from arrival of the patient in the consulting room to creation of the patient report, or to completion of the clinic letter dictation at the end of the consultation. The data are reported in minutes as mean consultation time ± standard deviation.

Approval. According to institutional policy and the UK Health Departments' Research Ethics Service[20] this work met criteria for clinical service provision exempt from ethics review.

RESULTS

Utilisation of PowerForms. Among the 695 new-patient referrals who attended the cardiac outpatient clinic during the study period, 594 were seen by participating cardiologists. PowerForms were utilised in 360 (61%) of these patients, the rate increasing from 42% in June 2016 to 77% in January 2017. The average consultation time, measured in the sub-study of 44 patients, was 13.97 ± 3.5 minutes using the PowerForm (n=22) compared with 14.22 ± 2.95 minutes using conventional paper documentation (n=22). Individual consultation times are shown in additional table A3.

Patient reports. Highly structured patient reports (Figure 1) were made available upon electronic sign-off for immediate inspection by the referring GP using HIE technology. Electronic transmission of the patient report, tested in a sub-study of 64 consecutive patients, was successful, permitting direct DTS delivery into relevant primary care EMIS files in 29 cases. The remaining patients were referred from practices not currently configured for the DTS mode of transmission and their reports were delivered by NHS mail, either to the practice mailbox (n=22) or to a generic

For peer review only - http://bmjopen.bmj.com/site/about/guidelines.xhtml

mailbox for postal delivery (n=13).

Patient survey. 27 women and 26 men who had been given a copy of their patient reports after outpatient consultation completed the questionnaire. There were no refusals. Detailed responses are provided in additional material (table A2). 52 (98%) patients found the report "easy to follow" and 49 (92%) believed it helpful "to understand (their) medical condition". Importantly 49 (92%) patients considered availability of the patient report immediately after the consultation as either "somewhat more useful" or "much more useful" than waiting for the postal delivery of a conventional typed letter. Ordinal logistic regression analysis identified no significant relationships between patients' age and sex and their opinions about the utility of the patient report in comparison with the conventional clinic letter (p>0.05) (table 1).

		95% Confidence Interval		
	Odds Ratio	Lower bound	Upper bound	p value
Age	1.00	0.52	1.92	1.00
Male (Ref = Female)	0.85	0.27	2.73	0.79

Table 1. Ordinal logistic regression analysis exploring the relationship between patient demographic variables and their thoughts on the utility of the patient report in comparison with the conventional dictated clinic letter (n=53).

General practitioner survey. Of the 210 GPs who were supplied with anonymised paired samples of patient reports and dictated letters, 93 (44%) responded to the survey. We were unable to obtain responses from the remainder of the GPs despite

For peer review only - http://bmjopen.bmj.com/site/about/guidelines.xhtml

sending further e-mail reminders. Detailed responses are provided in additional material (table A1). 74 (80%) GPs found the patient report “easy to follow”, while 69 (74%) considered it provided “adequate information for their clinical needs”. Electronic transmission into the patient’s EMIS file immediately after the consultation was considered important by 75 (81%) GPs, 41 rating it “extremely important” and 34 “very important”. 70 (75%) GPs found the new patient report more useful than the conventional typed letter, 19 rating it as “somewhat more useful” and 51 as “much more useful” (Figure 2).

Data extraction and aggregate analyses. Extraction of data entered into the PowerForm was successful for all 360 patients, confirming availability of these routinely collected outpatient data for audit and research. Sample analyses of reasons for outpatient referral, diagnostic categories and disposal decisions are presented in Figure 3.

DISCUSSION

This study has shown how development of a PowerForm tailored to the requirements of a specialist outpatient service facilitates the capture of routinely collected clinical data within the Cerner EHR. These data can be made available for audit and research. They can also enhance communication with primary care physicians and patients by automatically populating structured reports for immediate electronic delivery to the relevant EMIS files and for presentation to patients at the end of the consultation. The clinical utility of these reports was reflected in the surveys we conducted which documented high approval ratings from both primary care physicians and patients.

For peer review only - http://bmjopen.bmj.com/site/about/guidelines.xhtml

PowerForm development within the Cerner EHR provides templates for purposive data entry to meet the requirements of specific clinical tasks. In the present study we have responded to NHS Digital's ambition for a paperless NHS[21] by designing a PowerForm for routinely collected clinical data in the cardiology outpatient setting that automatically populates a SmartTemplate in producing a novel structured report. The immediate availability of the report at the end of the consultation allows a printed copy to be given to the patient as a record of the diagnosis, treatment and further management plan. Our survey showed the value patients placed on this prompt take-home communication that empowered them in helping to understand their medical condition. However, it is the electronic transmission methodology developed as part of this study that represents a true step forward in meeting the ambition for a paperless NHS. In contrast to the traditional clinic letter which may take several days to be typed and mailed to the referring GP, the computer-generated report can now be delivered into the relevant primary care EMIS file before the patient has left the outpatient department. This was seen as being either very important or extremely important by nearly 90% of GPs who completed the survey. Also favoured was the layout of the report and its structured content, standardising the communication of outpatient findings to the referring GP. Indeed, levels of satisfaction with the patient report were high across a variety of domains and it was a major finding in the present study that the majority of patients and GPs found it more useful than the traditional dictated clinic letter for communicating the findings of the outpatient consultation.

Use of the PowerForm did not prolong consultation times compared with paper-based consultation. Although there is likely to be a learning curve (this was not tested), our data showed that utilisation of the PowerForm by participating consultants increased

For peer review only - http://bmjopen.bmj.com/site/about/guidelines.xhtml

during the course of the study, perhaps reflecting increasing familiarity with its application and an understanding of its added value for communication with primary care physicians and patients. For a consultant practiced in the use of the PowerForm consultation times were unaffected, and this may help allay concerns that use of the EHR for recording clinical data is overly time consuming[4]. Indeed, it is possible to argue that time efficiency is enhanced when the time taken to type, review and mail dictated clinic letters is taken into account. Nevertheless, it should be recognised that comfort using computer systems is variable and the consultation times recorded in this study are not necessarily generalisable to all clinicians.

The clinical implications of our study are considerable because the technology applied in developing PowerForm and electronic transmission of the patient reports is potentially transportable and available for use in other non-cardiological outpatient settings. Wider development of PowerForms, or their equivalent in other hospital systems, will build up the EHR and provide a substantial data resource for audit and research. Our study confirmed that routinely collected data can be downloaded, anonymised and made available for analysis. The exciting research potential of the EHR has been widely reported[22-26] but its clinical audit function should not be overlooked. The audit exemplars we report - including reasons for outpatient referral, diagnostic categories and disposal decisions - appear mundane but this obscures the fact that information of this sort is almost never available to clinicians or hospital managers, particularly in settings where clinical documentation is paper-based or by free-text entry into computer systems. Understanding these patient profiles seems a basic requirement for effective organisation of outpatient services and provides a

For peer review only - http://bmjopen.bmj.com/site/about/guidelines.xhtml

compelling rationale for further developing the EHR as a repository for routinely collected clinical data.

It was a limitation of this study that the GPs we surveyed were restricted to one clinical commissioning group in East London and the response rate of 44% leaves the results prone to response bias. As such, the generalisability of our findings will need testing in larger groups. The same can be said of our convenience sample of 53 patients approached at the end of their outpatient consultation. Whilst there were no refusals, it is important to be aware that these highly positive responses were obtained from a relatively small patient group. A further limitation was the setting of the study in a general cardiology outpatient clinic and although it is likely that the technology is transportable to other clinical settings, this will need confirmation in future studies. Future studies of live use in other clinical settings will also allow further evaluation of the added value offered by the PowerForm.

CONCLUSIONS

Using a purpose built PowerForm, an automated patient report of a cardiac outpatient consultation can be developed providing a feasible alternative to the dictated clinic letter. This report is highly valued by both patients and GPs and is immediately available to both groups. The PowerForm encourages physicians to populate the EHR with coded data during real-time consultation. These data can then be de-identified, downloaded and used for audit and research. Implementation of the PowerForm across other specialties will allow healthcare professionals to capitalise on the benefits offered by the EHR.

For peer review only - http://bmjopen.bmj.com/site/about/guidelines.xhtml

LIST OF ABBREVIATIONS

EHR = Electronic Health Record, GP = General Practitioner, HIE = Health Information Exchange, EMIS = Egton Medical Information Systems, DTS = Data Transfer Service

DECLARATIONS

Ethics approval and consent to participate

Policy criterion: The work is primarily intended to improve local care, not provide generalizable knowledge in a field of inquiry.

Explanation: The work reported here meets this criterion because recording of outpatient data is a universally recommended practice. We sought only to evaluate improvements in recording of outpatient data using an electronic template and the advantages it presented in terms of communicating with primary care and providing data of potential value for audit and research.

Funding statement

This study was funded by The Guttmann Academic Partnership hosted by UCLPartners. NB had financial support from the Isaac Schapera Trust Fund for the submitted work. AML acknowledges support from the NIHR Cardiovascular Biomedical Research Unit at Barts Health NHS Trust and from the “SmartHeart” EPSRC programme grant (EP/P001009/1). AT and MRB acknowledge funding from the Medical Research Council (MR/K006584/1).

Data sharing statement

All data generated or analysed during this study are included in this published article [and its supplementary information files].

For peer review only - http://bmjopen.bmj.com/site/about/guidelines.xhtml

Competing interests

The authors declare that they have no competing interests.

Authors' contributions

NB designed data collection tools, monitored data collection for the whole study, wrote the statistical analysis plan, cleaned and analysed the data, and drafted and revised the paper. RAA designed data collection tools (creation of the PowerForm and utilisation over the period of the study) and cleaned and analysed the data. RW designed data collection tools (creation of the PowerForm and utilisation over the period of the study) and cleaned and analysed the data. MKDH designed data collection tools (surveys of feedback) and cleaned and analysed the data. MRB designed data collection tools and cleaned and analysed the data. AML designed data collection tools (download of data from PowerForms) and cleaned and analysed the data. SJ designed data collection tools (creation of PowerForm and SmartTemplate) and cleaned and analysed the data. CG designed data collection tools (creation of PowerForm and SmartTemplate) and cleaned and analysed the data. JR designed data collection tools and cleaned and analysed the data. AT designed data collection tools, monitored data collection for the whole study, cleaned and analysed the data, and drafted and revised the paper. He is guarantor. All authors provided intellectual input into the draft of the manuscript, and read, edited and approved the final version.

Acknowledgements

MKDH was in part supported by the National Institute for Health Research (NIHR) Collaboration for Leadership in Applied Health Research and Care (CLAHRC) North Thames at Bart's Health NHS Trust. AT acknowledges support of Barts

For peer review only - http://bmjopen.bmj.com/site/about/guidelines.xhtml

Cardiovascular Biomedical Research Unit, funded by the National Institute for Health Research.

REFERENCES

1. Gunter TD, Terry NP. The Emergence of National Electronic Health Record Architectures in the United States and Australia: Models, Costs, and Questions. J Med Internet Res. 7. Centre for Global eHealth Innovation, Toronto, Canada. 2005.

2. Yamamoto LG, Khan AN. Challenges of electronic medical record implementation in the emergency department. Pediatr Emerg Care. 2006;22(3):184-91; quiz 92.

3. Jha AK, DesRoches CM, Campbell EG, Donelan K, Rao SR, Ferris TG, et al. Use of electronic health records in U.S. hospitals. N Engl J Med. 2009;360(16):1628-38.

4. Kruse CS, Kristof C, Jones B, Mitchell E, Martinez A. Barriers to Electronic Health Record Adoption: a Systematic Literature Review. J Med Syst. 2016;40(12):252.

5. Wells BJ, Chagin KM, Nowacki AS, Kattan MW. Strategies for handling missing data in electronic health record derived data. EGEMS (Wash DC). 2013;1(3):1035.

6. Sterne JA, White IR, Carlin JB, Spratt M, Royston P, Kenward MG, et al. Multiple imputation for missing data in epidemiological and clinical research: potential and pitfalls. Bmj. 2009;338:b2393.

7. Rawal J, Barnett P, Lloyd BW. Use of structured letters to improve communication between hospital doctors and general practitioners. Bmj. 1993;307(6911):1044.

For peer review only

For peer review only - http://bmjopen.bmj.com/site/about/guidelines.xhtml

8. Hook SE, Banister GC, Topliss C, Webb J. Letters and notes in orthopaedic surgery. Ann R Coll Surg Engl. 2006;88(3):292-6.

9. Parks T, Kingham E, McEwen D, Cooper S. The preference of general practitioners for structured outpatient clinic letters. Clin Med (Lond). 2011;11(2):205-6.

10. Peridis S, Roberts D, Hopkins C. Outpatient clinic letters in ENT. Is there any margin of improvement? B-ent. 2012;8(1):33-5.

11. Blumenthal D, Tavenner M. The "meaningful use" regulation for electronic health records. N Engl J Med. 2010;363(6):501-4.

12. Buntin MB, Jain SH, Blumenthal D. Health information technology: laying the infrastructure for national health reform. Health Aff (Millwood). 2010;29(6):1214-9.

13. Centers for Medicare and Medicaid Services. Stage 2 Eligible Professional Meaning Use Core Measures Measure 8 of 17. 2012; 1–3.

14. Federman AD, Sanchez-Munoz A, Jandorf L, Salmon C, Wolf MS, Kannry J. Patient and clinician perspectives on the outpatient after-visit summary: a qualitative study to inform improvements in visit summary design. J Am Med Inform Assoc. 2016.

15. Cerner Millenium. About Cerner. http://www.cerner.com/About_Cerner. Accessed 16th February 2017.

16. Cerner Millenium. Global Overview. http://www.cerner.com/About_Cerner/Corporate_Profile. Accessed 16th February 2017.

17. Revised Standards for Quality Improvement Reporting Excellence SQUIRE 2.0. http://www.squire-statement.org. Accessed 1st April 2017.

For peer review only

18. Collier, Ken W. (2011). Agile Analytics: A Value-Driven Approach to Business Intelligence and Data Warehousing. Pearson Education. pp. 121 ff. ISBN 9780321669544. What is a self-organizing team?

19. Data Transfer Service. IBM Bluemix. URL: https://knowledgelayer.softlayer.com/topic/data-transfer-service. Accessed 17th February 2017.

20. United Kingdom Health Departments' Research Ethics Service. URL: http://www.hra.nhs.uk/documents/2016/06/defining-research.pdf. Accessed 24th August 2017

21. NHS England. Five Year Forward View. NHS England; 2014. URL: www .england.nhs.uk/wp-content/uploads/2014/10/5yfv-web.pdf. Accessed 17th February 2017.

22. Dean BB, Lam J, Natoli JL, Butler Q, Aguilar D, Nordyke RJ. Review: use of electronic medical records for health outcomes research: a literature review. Med Care Res Rev. 2009;66(6):611-38.

23. Cowie MR, Blomster JI, Curtis LH, Duclaux S, Ford I, Fritz F, et al. Electronic health records to facilitate clinical research. Clin Res Cardiol. 2017;106(1):1-9.

24. Cederholm S, Hill G, Asiimwe A, Bate A, Bhayat F, Persson Brobert G, et al. Structured assessment for prospective identification of safety signals in electronic medical records: evaluation in the health improvement network. Drug Saf. 2015;38(1):87-100.

25. Goldstein BA, Navar AM, Pencina MJ, Ioannidis JP. Opportunities and challenges in developing risk prediction models with electronic health records data: a systematic review. J Am Med Inform Assoc. 2017;24(1):198-208.

26. Denaxas SC, Morley KI. Big biomedical data and cardiovascular disease

For peer review only - http://bmjopen.bmj.com/site/about/guidelines.xhtml

research: opportunities and challenges. Eur Heart J Qual Care Clin Outcomes 2015;1: 9-16.

Legends for illustrations

Figure 1. Sample patient report: Sides 1 and 2

Figure 2. GP questionnaire. Responses to “How would you rate the utility of this new Outpatient Report compared with the conventional typed letter posted to your practice?”

Figure 3. Aggregate analysis of data extracted from the PowerForm in 360 patients (A) Indications for outpatient referral (B) Diagnostic categories (C) Disposal decisions

ADDITIONAL MATERIAL

ADDITIONAL TABLE A1. Responses to GP questionnaires. File format: .doc

ADDITIONAL TABLE A2. Responses to patient questionnaires. File format: .doc

ADDITIONAL TABLE A3. Consultation times for patients seen in general cardiology clinics (A) Using the PowerForm and SmartTemplate (B) Using paper-based consultation and dictated clinic letter. File format: .doc

For peer review only

For peer review only - http://bmjopen.bmj.com/site/about/guidelines.xhtml

Side 1

Hospital Logo NHS
NHS Trust

Cardiac Outpatient Report New Appointment

Consultant: Dr John Smith **Date Seen:** 1 January 2000

**GP Name:** XXXXXXXX<br>**GP Address:**<br>XXXXXXXXXX<br>XXXXXXXXXX<br>XXXXXXXXXX	**Patient First Name:** XXXXXXX<br>**Patient Last Name:** XXXXXXX<br>**MRN:** 123456<br>**NHS No:** 123456<br>**Sex:** XXXXX<br>**DOB:** 01/01/2000<br>**Address:** XXXXXX

Primary Complaint: Ankle oedema
Secondary Complaint(s):

Risk Factors

**Coronary Disease**	Smoking - Never smoked, Hypertension
**Hypertension**	

Vital Signs

**Pulse Rate**	65 bpm	**Weight**	76 kg
**SBP**	199 mmHg	**BMI**	27.92
**DBP**	105 mmHg		

Prior Procedure

Date	Procedure
04-AUG-2016	Echocardiogram

Diagnosis

Date	Diagnosis	Confirmation	Ranking
04-JAN-2017	Aortic sclerosis	Confirmed	Primary

Other Problems

Date	Problems & Diagnoses - Lifelong	Confirmation	Status
01-JUN-2015	History of - hypercholesterolaemia	Confirmed	Active
01-JUN-2015	Hypertension	Confirmed	Active
01-JUN-2015	Osteoarthritis	Confirmed	Active
01-JUN-2015	Coordinated support plan	Confirmed	Active

Side 2

Investigations Ordered (Find results on Cerner portal)

**ECG Test**	ECG Recorded
**ECG Result**	1° block, LVH
**Blood Tests**	
**Imaging**	
**Ambulatory monitoring**	
**Stress Testing**	

Treatment

**Treatment Status**		
**Drug**	**Dose & Frequency**	**Drug Change**
Atorvastatin (Lipitor)	40mg Once Per Day	
Furosemide	40mg Once Per Day	
Candesartan	8mg Once Per Day	Dose change
Bisoprolol	2.5mg Once Per Day	
Nifedipine	60mg Once Per Day	
Iron Replacement	Twice Per Day	

Disposal

**Further Management**	Discharge to GP
**Waiting List:**	
**Specialist Clinic:**	
**Comments to GP:**	Ankle oedema resolved<br>No evidence of CCF
**Action points to GP:**	BP too high - suggest push up candesartan to 8mg daily while monitoring renal function

Figure 1. Sample patient report: Sides 1 and 2

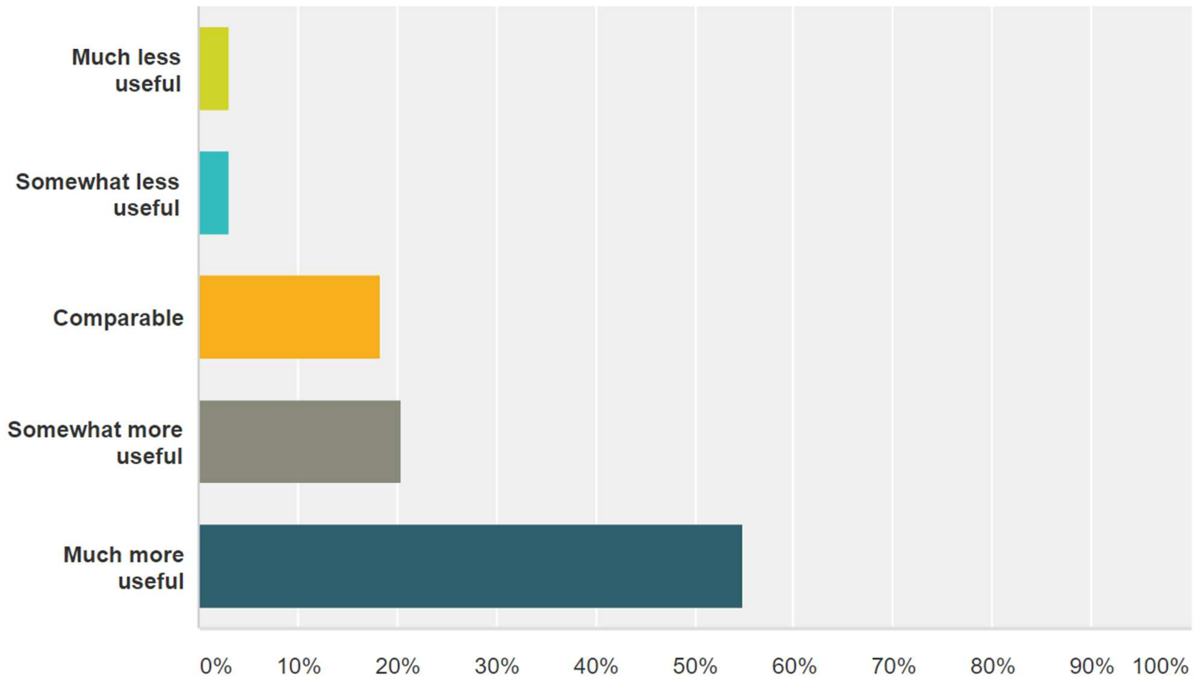

Figure 2. GP questionnaire. Responses to "How would you rate the utility of this new Outpatient Report compared with the conventional typed letter posted to your practice?"

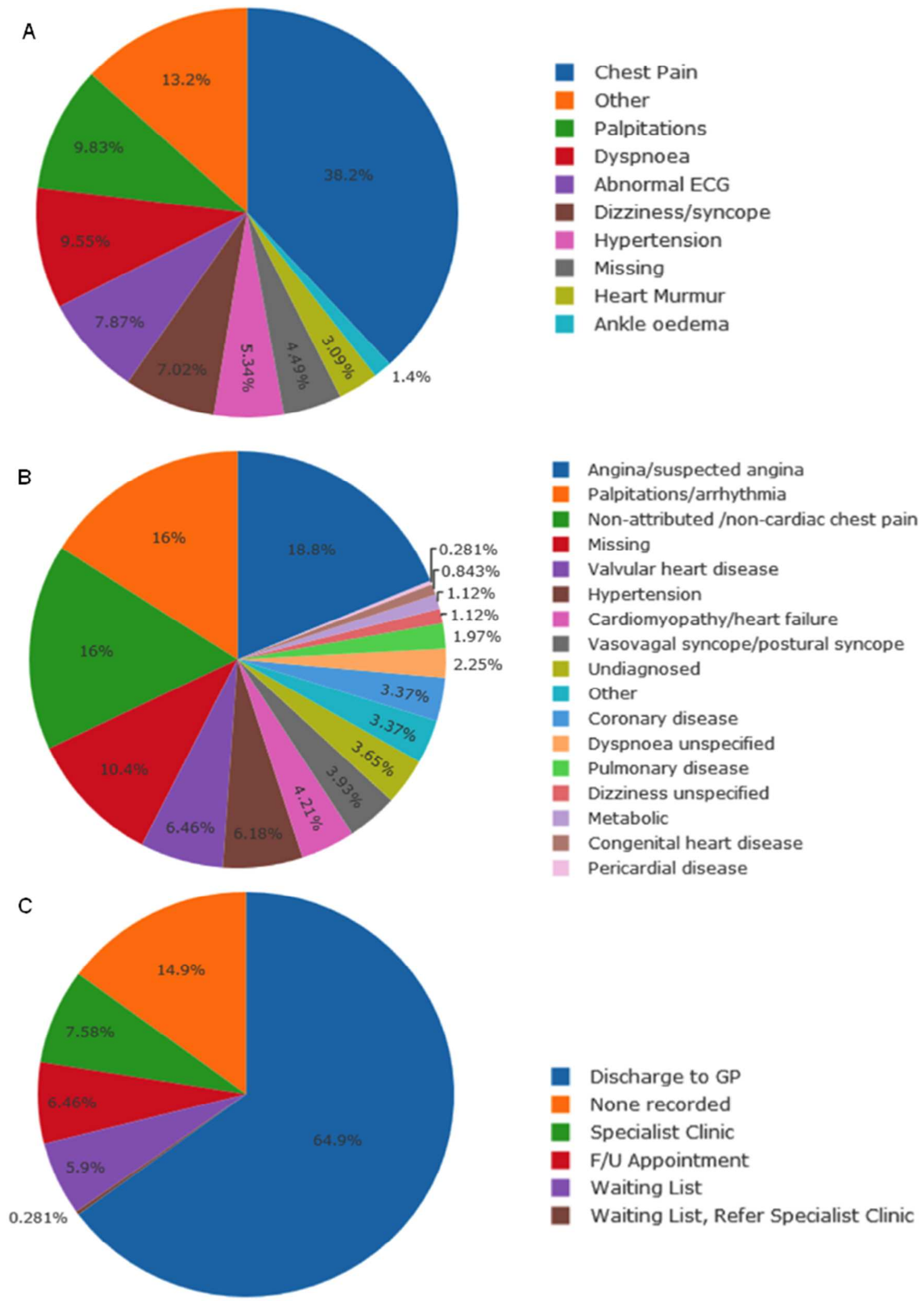

Figure 3. Aggregate analysis of data extracted from the PowerForm in 360 patients (A) Indications for outpatient referral (B) Diagnostic categories (C) Disposal decisions

ADDITIONAL TABLE A1. Responses to GP questionnaires

Questions (posed to 93 GPs)	Cardiac outpatient report questionnaire for GPs				
How many GPs work in your practice? (93 responses)	**0-1**	**2-4**	**5+**		
	3	34	56		
	3.2%	36.6%	60.2%		
The patient is presented with a copy of the Outpatient Report on leaving the cardiac outpatient department. How important do you think this is? (93 responses)	**Not at all important**	**Slightly important**	**Somewhat important**	**Very important**	**Extremely important**
	0	0	15	48	30
	0.0%	0.0%	16.1%	51.6%	32.3%
The Outpatient Report is transmitted electronically to the patient's EMIS file immediately after the consultation. How important do you think same-day delivery of the Outpatient Report is? (89 responses)	**Not at all important**	**Slightly important**	**Somewhat important**	**Very important**	**Extremely important**
	0	4	10	34	41
	0.0%	4.5%	11.2%	38.2%	46.1%
The SNOMED codes used in the Outpatient Report will permit the transfer of data directly into the relevant EMIS fields. How useful do you think this will be? (92 responses)	**Not at all useful**	**Slightly useful**	**Somewhat useful**	**Very useful**	**Extremely useful**
	1	2	9	37	43
	1.1%	2.2%	9.8%	40.2%	46.7%
Is the layout of the Outpatient Report easy to follow? (89 responses)	**Yes**	**No**	**Somewhat**		
	74	2	13		
	83.1%	2.2%	14.6%		
Does the content of the Outpatient Report provide adequate information for your clinical needs? (92 responses)	**Yes**	**No**	**Somewhat**		
	69	5	18		
	75.0%	5.4%	19.6%		
The Outpatient Report provides a list of investigations and medications that a patient will receive after their clinic appointment. How useful do you think these sections are for your further follow up of patients? (88 responses)	**Not at all useful**	**Slightly useful**	**Somewhat useful**	**Very useful**	**Extremely useful**
	0	0	6	35	47
	0.0%	0.0%	6.8%	39.8%	53.4%
How would you rate the utility of this new Outpatient Report compared with the conventional typed letter posted to your practice? (93 responses)	**Much less useful**	**Somewhat less useful**	**Comparable**	**Somewhat more useful**	**Much more useful**
	3	3	17	19	51
	3.2%	3.2%	18.3%	20.4%	54.8%

For peer review only

ADDITIONAL TABLE A2. Responses to patient questionnaires

Questions (posed to 53 Patients)	Cardiac outpatient report questionnaire for patients				
Which age group are you a part of? (53 responses)	**16-35**	**36-55**	**56-75**	**76+**	**Prefer not to say**
	7	14	24	8	0
	13.2%	26.4%	45.3%	15.1%	0.0%
What is your gender? (53 responses)	**Male**	**Female**	**Prefer not to say**		
	26	27	0		
	49.1%	50.9%	0.0%		
As a patient, you are given a copy of the Outpatient Report on leaving the cardiac outpatient department. How important do you think this is? (53 responses)	**Not at all important**	**Slightly important**	**Somewhat important**	**Very important**	**Extremely important**
	1	0	3	29	20
	1.9%	0.0%	5.7%	54.7%	37.7%
Is the layout of the Outpatient Report easy to follow? (53 responses)	**Yes**	**No**	**Somewhat**		
	52	0	1		
	98.1%	0.0%	1.9%		
How would you rate this new Outpatient Report compared with the conventional typed letter posted to your home? (53 responses)	**Much less useful**	**Somewhat less useful**	**Comparable**	**Somewhat more useful**	**Much more useful**
	0	0	4	12	37
	0.0%	0.0%	7.5%	22.6%	69.8%
Do you think this Outpatient Report will help you to understand your medical condition? (53 responses)	**Yes**	**No**	**Somewhat**		
	49	1	3		
	92.5%	1.9%	5.7%		
Does the Outpatient Report help you understand the follow up you will receive after your consultation? (53 responses)	**Yes**	**No**	**Somewhat**		
	49	3	1		
	92.5%	5.7%	1.9%		

For peer review only

ADDITIONAL TABLE A3. Consultation times for patients seen in general cardiology clinics (A) Using the PowerForm and SmartTemplate (B) Using paper-based consultation and dictated clinic letter.

	(A) SmartTemplate		(B) Dictated Clinic Letter	
	Patient	Consultation Time (minutes: seconds)	Patient	Consultation Time (minutes: seconds)
	1	11:03	1	14:07
	2	15:03	2	18:48
	3	23:26	3	15:57
	4	14:41	4	11:33
	5	10:22	5	12:57
	6	15:21	6	13:59
	7	14:36	7	19:14
	8	11:28	8	15:27
	9	14:41	9	11:12
	10	16:53	10	10:48
	11	11:21	11	10:11
	12	12:02	12	12:38
	13	08:29	13	13:36
	14	12:28	14	13:26
	15	20:01	15	12:41
	16	14:16	16	21:13
	17	12:34	17	12:05
	18	12:35	18	16:27
	19	11:36	19	10:53
	20	19:27	20	14:09
	21	11:26	21	13:50
	22	13:34	22	17:43

For peer review only

**Mean consultation time**		13:58		14:13
**Standard deviation**		03:30		02:57

For peer review only

Text section and item name	Section or item description	Included?		Page number
Notes to authors	‣ The SQUIRE guidelines provide a framework for reporting new knowledge about how to improve healthcare.			
	‣ The SQUIRE guidelines are intended for reports that describe system level work to improve the quality, safety and value of healthcare, and used methods to establish that observed outcomes were due to the intervention(s).			
	‣ A range of approaches exists for improving healthcare. SQUIRE may be adapted for reporting any of these.			
	‣ Authors should consider every SQUIRE item, but it may be inappropriate or unnecessary to include every SQUIRE element in a particular manuscript.			
	‣ The SQUIRE glossary contains definitions of many of the key words in SQUIRE.			
	‣ The explanation and elaboration document provides specific examples of well-written SQUIRE items and an in-depth explanation of each item.			
	‣ Please cite SQUIRE when it is used to write a manuscript.			
Title and abstract				
1. Title	Indicate that the manuscript concerns an initiative to improve healthcare (broadly defined to include the quality, safety, effectiveness, patient-centredness, timeliness, cost, efficiency and equity of healthcare).	Yes	Discussed that we are enhancing communication to improve healthcare	0
2. Abstract	a. Provide adequate information to aid in searching and indexing.	Yes		1

	b. Summarise all key information from various sections of the text using the abstract format of the intended publication or a structured summary such as: background, local problem, methods, interventions, results, conclusions.	Yes		1
Introduction	*Why did you start?*			
3. Problem description	Nature and significance of the local problem.	Yes	EHR under-utilised. OP consultations don't contribute. Communication difficulties with primary care & patient.	2+3
4. Available knowledge	Summary of what is currently known about the problem, including relevant previous studies.	Yes	AVS used in USA. No studies in cardiology.	3+4
5. Rationale	Informal or formal frameworks, models, concepts and/or theories used to explain the problem, any reasons or assumptions that were used to develop the intervention(s) and reasons why the intervention(s) was expected to work	Yes	SNOMED-based electronic PowerForm comprising a user-friendly interface for real-time entry of clinical data into Millennium during consultation in a general cardiac	3+4

			outpatient clinic	
6. Specific aims	Purpose of the project and of this report.	Yes		4
Methods	*What did you do?*			
7. Context	Contextual elements considered important at the outset of introducing the intervention(s).	Yes	Description of Cerner. Also discussed that this was used in general cardiology clinics at Barts Health NHS Trust	3+4
8. Intervention(s)	a. Description of the intervention(s) in sufficient detail that others could reproduce it.	Yes	Development of PowerForm, smart template, transfer + data download methodology	4+5
	b. Specifics of the team involved in the work.	Yes	3 cardiologists	4+5
9. Study of the intervention(s)	a. Approach chosen for assessing the impact of the intervention(s).	Yes	Electronic transfer, data download	4+5
	b. Approach used to establish whether the observed outcomes were due to the intervention(s).	Yes	Data download, electronic transfer, satisfaction	4-8

			surveys, consultation times	
10. Measures	a. Measures chosen for studying processes and outcomes of the intervention(s), including rationale for choosing them, their operational definitions and their validity and reliability.	Yes	Surveys of feedback, download of data, electronic transfer, consultation times	4-8
	b. Description of the approach to the ongoing assessment of contextual elements that contributed to the success, failure, efficiency and cost.	Yes		
	c. Methods employed for assessing completeness and accuracy of data.	Yes		
11. Analysis	a. Qualitative and quantitative methods used to draw inferences from the data.	Yes	Ordinal logistic regression, mean consultation times ± SD	9
	b. Methods for understanding variation within the data, including the effects of time as a variable.	Yes	Used ordinal logistic regression to determine relationship between patient demographics & utility of report versus dictated letter	9

12. Ethical considerations	Ethical aspects of implementing and studying the intervention(s) and how they were addressed, including, but not limited to, formal ethics review and potential conflict(s) of interest.	Yes	Ethics statement, patient/GP surveys anonymised & voluntary	8
Results	*What did you find?*			
13. Results	a. Initial steps of the intervention(s) and their evolution over time (eg, time-line diagram, flow chart or table), including modifications made to the intervention during the project.	Yes	Usage of the PowerForm over an 8 month time period	10
	b. Details of the process measures and outcomes.	Yes	Usage of PowerForm, data download, electronic transfer, consultation times, feedback	10
	c. Contextual elements that interacted with the intervention(s).	Yes	Ordinal logistic regression for patient age/gender & thought's on utility of OP report	9
	d. Observed associations between outcomes, interventions and relevant contextual elements.	Yes	No association between patient	9

			demographics & thoughts on utility of OP report	
	e. Unintended consequences such as unexpected benefits, problems, failures or costs associated with the intervention(s).	Yes	Effect of PowerForm use on consultation times. Also examined electronic transfer capability.	8+9
	f. Details about missing data.	Yes	Addressed that we had a low response rate from GPs and that patient sample size was small	13
Discussion	*What does it mean?*			
14. Summary	a. Key findings, including relevance to the rationale and specific aims.	Yes	First paragraph of discussion	10
	b. Particular strengths of the project.	Yes	Helps the NHS become paperless, data download fully successful and patient/GP	10-12

			satisfaction very high	
15. Interpretation	a. Nature of the association between the intervention(s) and the outcomes.	Yes	Intervention enabled data download and electronic transfer. Intervention was also highly rated by GPs & patients. Slight prolongation in clinic times.	10-13
	b. Comparison of results with findings from other publications.	Yes	Discussed how our findings can be used to improve the repository of data within the HER	12
	c. Impact of the project on people and systems.	Yes	Use in audit/research	12
	d. Reasons for any differences between observed and anticipated outcomes, including the influence of context.	Yes	Consultant uptake increased over the 8 month time period, perhaps	12

			reflecting increased familiarity with the software	
	e. Costs and strategic trade-offs, including opportunity costs.	Yes	Despite prolonged consultation times, PowerForm may cut overall time taken to dictate a clinic letter	12
16. Limitations	a. Limits to the generalisability of the work.	Yes	Low response rate, cardiac outpatient setting	13
	b. Factors that might have limited internal validity such as confounding, bias or imprecision in the design, methods, measurement or analysis.	Yes	Discussed that patient sample was a convenience sample. Also discussed that our consultation time findings were found with a cardiologist who was	13

			familiar with the PowerForm	
	c. Efforts made to minimise and adjust for limitations.	Yes	PowerForms were available to 3 participating cardiologists	5
17. Conclusions	a. Usefulness of the work.	Yes	Discussed that enables data download, direct electronic transfer. Also discussed that patient report is highly valued by patients and GPs.	13
	b. Sustainability.	Yes	Described how the PowerForm is a feasible alternative to the dictated clinic letter. Also described that we used it over an 8 month time period	13

	c. Potential for spread to other contexts.	Yes	Other specialties	12+13
	d. Implications for practice and for further study in the field.	Yes	Low GP response rate mentioned as further studies in this group. Also implications for audit/research & communication	12+13
	e. Suggested next steps.	Yes	Discussion about how integration will allow us to benefit from EHR	13
Other information				
18. Funding	Sources of funding that supported this work. Role, if any, of the funding organisation in the design, implementation, interpretation and reporting.	Yes	Mentioned during online submission process & funding statement	14

BMJ Open

Feasibility of real-time capture of routine clinical data in the electronic health record: a hospital based, observational service-evaluation study

Journal:	*BMJ Open*
Manuscript ID	bmjopen-2017-019790.R1
Article Type:	Research
Date Submitted by the Author:	07-Dec-2017

Complete List of Authors:	Bodagh, Neil; Barts Health NHS Trust, Barts Heart Centre<br>Archbold, R; Barts Health NHS Trust, Barts Heart Centre<br>Weerackody, Roshan; Barts Health NHS Trust, Barts Heart Centre<br>Hawking, Meredith; Barts and The London School of Medicine and Dentistry, Centre for Primary Care and Public Health<br>Barnes, Michael; William Harvey Research Institute, Director of Bioinformatics<br>Lee, Aaron; William Harvey Research Institute, Centre for Advanced Cardiovascular Imaging<br>Janjuha, Surjeet; Barts Health NHS Trust, Electronic Health Record Development Manager<br>Gutteridge, Charles; Barts Health NHS Trust,<br>Robson, John; Barts and The London School of Medicine and Dentistry, Queen Mary University of London, Centre for Primary Care and Public Health<br>Timmis, Adam; Bart's Heart Centre, NIHR Cardiovascular Biomedical Research Unit; University College London, Farr Institute

**Primary Subject Heading**:	Health informatics
Secondary Subject Heading:	Cardiovascular medicine
Keywords:	Health informatics < BIOTECHNOLOGY & BIOINFORMATICS, CARDIOLOGY, Information technology < BIOTECHNOLOGY & BIOINFORMATICS

SCHOLARONE™
Manuscripts

Feasibility of real-time capture of routine clinical data in the electronic health record: a hospital based, observational service-evaluation study

Neil Bodagh MB ChB[1], R. Andrew Archbold MD[1], Roshan Weerackody PhD[1], Meredith K. D. Hawking MPH[2], Michael R Barnes PhD[3], Aaron M Lee PhD[4], Surjeet Janjuha HND[5], Charles Gutteridge FRCP[6], John Robson FRCGP[2] , Adam Timmis MD[1,7]

[1] Barts Heart Centre, London, UK
[2] Clinical Effectiveness Group, Queen Mary University of London, UK
[3] Director of Bioinformatics, William Harvey Research Institute Queen Mary University of London, UK
[4] Centre for Advanced Cardiovascular Imaging, William Harvey Research Institute, Queen Mary University of London, UK
[5] EHR Development Manager, Barts Health NHS Trust, London, UK
[6] Chief Clinical Information Officer, Barts Health NHS Trust, London, UK
[7] The Farr Institute of Health Informatics Research, University College London, UK

Corresponding author: Dr Neil Bodagh, Barts Heart Centre, W Smithfield, London EC1A 7BE, Tel +447774836031, Fax +44 20 3416 5000, Email: neil.bodagh@nhs.net

KEY WORDS

General Practitioners, Health Information Exchange, Outpatients, Primary Health Care, Surveys and Questionnaires

Word count: 3154

For peer review only

For peer review only - http://bmjopen.bmj.com/site/about/guidelines.xhtml

ABSTRACT

Objectives. The electronic health record (EHR) is under-utilised in the hospital setting. The aim of this service evaluation study was to respond to NHS Digital's ambition for a paperless NHS by capturing routinely collected cardiac outpatient data in the EHR to populate summary patient reports and provide a resource for audit and research.

Design. A PowerForm template was developed within the Cerner EHR, for real-time entry of routine clinical data by clinicians attending a cardiac outpatient clinic. Data captured within the PowerForm automatically populated a SmartTemplate to generate a view-only report that was immediately available for the patient and for electronic transmission to the referring general practitioner (GP).

Results. During the first eight months, the PowerForm template was used in 61% (360/594) of consecutive outpatient referrals increasing from 42% to 77% during the course of the study. Structured patient reports were available for immediate sharing with the referring GP using Cerner Health Information Exchange technology while electronic transmission was successfully developed in a sub-study of 64 cases, with direct delivery by the NHS Data Transfer Service in 29 cases and NHS mail in the remainder. In feedback, the report's immediate availability was considered very or extremely important by >80% of the patients and GPs who were surveyed. Both groups reported preference of the patient report to the conventional typed letter. De-identified template data for all 360 patients were successfully captured within the Trust system, confirming availability of these routinely collected outpatient data for audit and research.

For peer review only - http://bmjopen.bmj.com/site/about/guidelines.xhtml

Conclusion. Electronic template development tailored to the requirements of a specialist outpatient clinic facilitates capture of routinely collected data within the Cerner EHR. These data can be made available for audit and research. They can also be used to enhance communication by populating structured reports for immediate delivery to patients and GPs.

For peer review only

STRENGTHS AND LIMITATIONS

- Digital templates for data capture in the electronic health record (EHR) were tested in real-time during routine outpatient consultations confirming their clinical practicality.
- The potential utility of data captured within the templates for audit and research was demonstrated by successful download and aggregated analysis of an anonymised extract.
- Methodology was developed for immediate generation of an outpatient report and its electronic transfer to the referring general practitioner.
- The utility of the outpatient report was examined in a survey of general practitioners
- It was a limitation that the GPs we surveyed were restricted to one clinical commissioning group in East London and the response rate of 44% leaves the results prone to response bias.

For peer review only - http://bmjopen.bmj.com/site/about/guidelines.xhtml

BACKGROUND

The electronic health record (EHR) is a longitudinal accumulation of electronic health information collected during routine healthcare provision[1]. It has the potential to optimise documentation of patient encounters, aid communication between healthcare professionals, improve access to medical information and form a repository of clinical data for use in audit and research[2]. Outpatient consultations are frequent hospital-based clinical interactions and represent an important missed opportunity to contribute clinical data to the EHR. Encounters are usually documented in a dictated clinic letter that is either stored in the paper record or scanned into an electronic repository where it cannot readily be searched or curated. The variable structure and content of the dictated clinic letter undermines its utility for communicating clinical information with previous studies affirming that general practitioners (GPs) prefer structured correspondence about the patients they refer for specialist outpatient care[3-6]. Communication is further undermined by the inherent inefficiency of the clinic letter which can be highly variable in the time taken to arrive at the address of the referring primary care physician after the index consultation. Appropriate utilisation of the EHR presents a possible solution to these problems. To our knowledge, the EHR's ability to automatically generate reports of the outcomes of outpatient encounters have not been examined in the United Kingdom.

Previous failures to deliver a nationwide EHR within the UK have resulted in severe under-utilisation within the National Health Service (NHS)[7]. Under-utilisation has been attributed to a variety of factors that include concerns about disruptions to workflow and difficulties with inputting medical record data[8]. The volume of missing data ensures that few audit and research outputs are based on routinely collected

For peer review only - http://bmjopen.bmj.com/site/about/guidelines.xhtml

data within the EHR[9, 10]. In an effort to rectify the issue, the NHS announced an ambition to become fully paperless by 2020[11]. Crucial to the fulfilment of this ambition will be the development and improvement of EHR systems.

In the USA, there has been a drive to increase utilisation of the EHR through the "EHR Meaningful Use Programme"[12, 13]. This has led to the development of an electronic after-visit summary which aims to provide patients and their referring clinicians with relevant information and actionable instructions[14]. Studies to date have demonstrated that the after-visit summary is valued by both patients and primary care physicians[15]. The EHR is now increasingly used to generate discharge summaries for patients who have received inpatient care[16] but its use in the outpatient setting for real time data capture and development of clinic letters has received little attention and we have identified only a single report from a cancer clinic[17]. As far as we are aware, the potential benefits of such a document in a cardiac outpatient setting have not been examined.

The CERNER Millennium (*Cerner Millenium, Kansas, USA*) EHR system operates in >20 hospital Trusts across the UK[18] and is widely installed globally[19]. In the present study we have used Cerner's PowerChart application to develop a SNOMED-based electronic PowerForm comprising a user-friendly interface for real-time entry of clinical data during consultation in a general cardiac outpatient clinic. The aims of this study were 1) to test the feasibility of outpatient data capture in digital format for automatic development of a structured patient report, 2) to examine the effects of PowerForm utilisation on consultation times, 3) to develop methods for immediate electronic delivery of patient reports to referring primary care physicians, 4) to

For peer review only - http://bmjopen.bmj.com/site/about/guidelines.xhtml

determine the value of patient reports for improving communication with patients and primary care physicians and 5) to confirm the availability of outpatient data entered into the PowerForm for audit and research.

METHODS

In presenting this research we have adhered to Standards for Quality Improvement Reporting Excellence (SQUIRE) guidelines for reporting new knowledge about how to improve healthcare[20].

PowerForm. Technical build experts used the Cerner PowerChart application to develop an electronic template (PowerForm) for clinical data entry according to a strict specification based on the following queries: reason for referral, presenting symptoms, risk factors for cardiovascular disease and hypertension, prior cardiac procedures, examination findings, investigations ordered, diagnosis and problems, cardiac treatment and discharge/follow-up arrangements. The queries were developed by a consultant cardiologist and then modified by consensus of the user group. In order to ensure faithful data entry, standardised responses to the PowerForm queries were listed in drop down menus or in tabular displays requiring single or multiple tick-box responses. An adaptation of Agile methodology[21] was used in developing the PowerForm in incremental steps which were each tested and modified as necessary before being added to the software bank that contributed to the final product. Ease of use was enhanced by applying conditional logic to guide data entry into those fields relevant for a particular patient. The data captured by the PowerForm populated some of the existing data fields within the EHR such as "Cardiac Procedures" and "Diagnosis and Problems" using SNOMED terms

For peer review only - http://bmjopen.bmj.com/site/about/guidelines.xhtml

throughout while the additional cardiac-specific information populated new fields, further enriching Cerner's digital data repository.

SmartTemplate. This was developed using the PowerChart application to pull information from the PowerForm into a highly structured "patient report" that summarised the key clinical findings. The report included the reason for referral, risk factors, vital signs, diagnosis and problems, investigations, treatment, discharge/follow-up arrangements and action points for the referring GP. Again, an adaptation of Agile methodology[21] was used in developing the SmartTemplate which was designed to replace the conventional dictated letter in providing the referring GP and the patient with necessary information about the clinic visit. The view-only report generated within the SmartTemplate was immediately available at the end of the consultation.

Participants. The outpatient PowerForm was made available to three consultant cardiologists attending a weekly general cardiology clinic in Barts Health NHS Trust. New referrals seen in the clinic from 1/6/2016 to 31/1/2017 were included. Follow-up patients were excluded. Use of the PowerForm was at the discretion of the participating consultants who had the alternative option of making a conventional paper record of the consultation and dictating a clinic letter to the referring GP. Data were entered into the PowerForm in real time and at the end of the consultation there was the opportunity to present the patient with a printed copy of the patient report.

Consultation times. These were calculated in a substudy of 44 new referrals seen by one of the participating consultants in seven consecutive clinics. The consultation

For peer review only - http://bmjopen.bmj.com/site/about/guidelines.xhtml

times for PowerForm consultations were compared with the consultation times taken for conventional paper consultations. Consultation time was defined as the time from arrival of the patient in the consulting room to creation of the patient report, or to completion of the clinic letter dictation at the end of the consultation. Consultation times were manually collected using a stopwatch. The data are reported in minutes as mean consultation time ± standard deviation.

Patient report delivery to GP. The patient report, generated within the SmartTemplate, was designed for electronic delivery to the referring GP. This function was introduced incrementally, starting with Cerner Health Information Exchange (HIE) technology to mirror the patient report across the interface between primary and secondary care. This allowed for inspection of the patient report by the GP in the patient's Egton Medical Information Systems (EMIS) file without true data export. The technology for exporting the patient reports directly into the primary care record was then developed and tested in a separate sample of 64 cardiac out-patients. The technology utilised the Data Transfer Service (DTS)[22] in 125 local practices that had been appropriately configured. Upon electronic sign-off of the report at the end of the consultation the DTS delivered it directly into the patient's EMIS file. In those local practices not yet configured for DTS transmission, the reports were delivered by NHS mail.

GP and patient surveys. In order to obtain feedback about the utility of the patient reports, 210 GPs in the Newham Clinical Commissioning Group were surveyed, using SurveyMonkey software (*SurveyMonkey Inc., Palo Alto, California, USA*). They were provided with three samples of anonymised patient reports and paired dictated

For peer review only - http://bmjopen.bmj.com/site/about/guidelines.xhtml

letters and invited to complete the survey by answering questions about their preferences. A paper-based survey was also conducted of 53 patients who were invited to answer questions after their consultation upon receipt of their patient report. Participation in both surveys was voluntary and all responses were anonymised. The survey questions are shown in additional tables A1 and A2. An ordinal logistic regression model was used to test whether or not a patient's gender or age group could predict a patient's thoughts on the utility of the patient report compared with the conventional dictated clinic letter. The data were analysed using SPSS for Windows v.17.0 software (*SPSS Inc., Chicago, IL, USA*).

Extraction of cardiac outpatient data. The clinical dataset recorded within the cardiac outpatient PowerForm was extracted from Trust Data Warehouse SQL server tables using a combination of queries. These data were password protected and stored within the NHS Trust system and were then anonymised. Identifying information was removed, including NHS identifiers, address details, visit dates, and the identification of medical staff and centres. Retained demographic data were limited to patient gender, age and ethnicity. These anonymised data were then subjected to the aggregated analysis presented in this work. Data manipulation was performed using the Python software language and visualised using the Plotly framework (*Plotly, Montreal, Quebec, Canada*).

Approval. According to institutional policy and the UK Health Departments' Research Ethics Service[23] this work met criteria for clinical service provision exempt from ethics review.

For peer review only - http://bmjopen.bmj.com/site/about/guidelines.xhtml

RESULTS

Utilisation of PowerForms. Among the 695 new-patient referrals who attended the cardiac outpatient clinic during the study period, 594 were seen by participating cardiologists. PowerForms were utilised in 360 (61%) of these patients, the rate increasing from 42% in June 2016 to 77% in January 2017. The average consultation time, measured in the sub-study of 44 patients, was 13.97 ± 3.5 minutes using the PowerForm (n=22) compared with 14.22 ± 2.95 minutes using conventional paper documentation (n=22). Individual consultation times are shown in additional table A3.

Patient reports. Highly structured patient reports (Figure 1) were made available upon electronic sign-off for immediate inspection by the referring GP using HIE technology. Electronic transmission of the patient report, tested in a sub-study of 64 consecutive patients, was successful, permitting direct DTS delivery into relevant primary care EMIS files in 29 cases. The remaining patients were referred from practices not currently configured for the DTS mode of transmission and their reports were delivered by NHS mail, either to the practice mailbox (n=22) or to a generic mailbox for postal delivery (n=13).

General practitioner survey. Of the 210 GPs who were supplied with anonymised paired samples of patient reports and dictated letters, 93 (44%) responded to the survey. We were unable to obtain responses from the remainder of the GPs despite sending further e-mail reminders. Detailed responses are provided in additional material (table A1). 74 (80%) GPs found the patient report "easy to follow", while 69 (74%) considered it provided "adequate information for their clinical needs". Electronic transmission into the patient's EMIS file immediately after the consultation

For peer review only

was considered important by 75 (81%) GPs, 41 rating it "extremely important" and 34 "very important". 70 (75%) GPs found the new patient report more useful than the conventional typed letter, 19 rating it as "somewhat more useful" and 51 as "much more useful" (Figure 2).

Patient survey. 27 women and 26 men who had been given a copy of their patient reports after outpatient consultation completed the questionnaire. There were no refusals. Detailed responses are provided in additional material (table A2). 52 (98%) patients found the report "easy to follow" and 49 (92%) believed it helpful "to understand (their) medical condition". Importantly 49 (92%) patients considered availability of the patient report immediately after the consultation as either "somewhat more useful" or "much more useful" than waiting for the postal delivery of a conventional typed letter. Ordinal logistic regression analysis identified no significant relationships between patients' age and sex and their opinions about the utility of the patient report in comparison with the conventional clinic letter ($p>0.05$) (table 1).

		95% Confidence Interval		
	Odds Ratio	Lower bound	Upper bound	p value
Age	1.00	0.52	1.92	1.00
Male (Ref = Female)	0.85	0.27	2.73	0.79

Table 1. Ordinal logistic regression analysis exploring the relationship between patient demographic variables and their thoughts on the utility of the patient report in comparison with the conventional dictated clinic letter (n=53).

For peer review only - http://bmjopen.bmj.com/site/about/guidelines.xhtml

Data extraction and aggregate analyses. Extraction of data entered into the PowerForm was successful for all 360 patients, confirming availability of these routinely collected outpatient data for audit and research. Sample analyses of reasons for outpatient referral, diagnostic categories and disposal decisions are presented in Figure 3.

DISCUSSION

This study has shown how development of a PowerForm tailored to the requirements of a specialist outpatient service facilitates the capture of routinely collected clinical data within the Cerner EHR. These data can be made available for audit and research. The PowerForm has the potential to enhance communication with primary care physicians and patients by automatically populating structured reports for immediate electronic delivery to the relevant EMIS files and for presentation to patients at the end of the consultation. The clinical utility of these reports was reflected in the surveys we conducted which documented high approval ratings from both primary care physicians and patients. In the present study we have responded to NHS Digital's ambition for a paperless NHS[11] by designing a PowerForm for routinely collected clinical data in the cardiology outpatient setting that automatically populates a SmartTemplate in producing a novel structured report. PowerForm development within the Cerner EHR provides templates for purposive data entry to meet the requirements of specific clinical tasks.

Our data showed that utilisation of the PowerForm by participating consultants increased during the course of the study, perhaps reflecting increasing familiarity with its application and an understanding of its added value for communication with

For peer review only - http://bmjopen.bmj.com/site/about/guidelines.xhtml

primary care physicians and patients. The low rate of PowerForm utilisation early after its introduction may have been attributable to an initial resistance to a change in work habit, a well-recognised barrier to EHR adoption[8]. Another barrier to EHR adoption is the time burden that is perceived to ensue from its use. However, we found that use of the PowerForm did not prolong consultation times compared with paper-based consultation. For a consultant practiced in the use of the PowerForm consultation times were unaffected, and this may help allay concerns that use of the EHR is overly time consuming. Indeed, it is possible to argue that time efficiency is enhanced when the time taken to type, review and mail dictated clinic letters is taken into account.

The immediate availability of the report at the end of the consultation allows a printed copy to be given to the patient as a record of the diagnosis, treatment and further management plan. However, it is the electronic transmission methodology developed as part of this study that represents a true step forward in meeting the ambition for a paperless NHS. In contrast to the traditional clinic letter which may take several days to be typed and mailed to the referring GP, the computer-generated report can now be delivered into the relevant primary care EMIS file before the patient has left the outpatient department.

Our survey showed the value patients placed on this prompt take-home communication that empowered them in helping to understand their medical condition. This was seen as being either very important or extremely important by nearly 90% of GPs who completed the survey. Also favoured was the layout of the report and its structured content, standardising the report of outpatient findings to the

For peer review only - http://bmjopen.bmj.com/site/about/guidelines.xhtml

referring GP. Indeed, levels of satisfaction with the patient report were high across a variety of domains and it was a major finding in the present study that the majority of patients and GPs found it more useful than the traditional dictated clinic letter for communicating the findings of the outpatient consultation.

Our study confirmed that routinely collected data can be downloaded, anonymised and made available for analysis. The exciting research potential of the EHR has been widely reported[24-28] yet at present the volume of missing data is a major barrier to its use[9, 10]. Our study has demonstrated that use of the PowerForm can help to overcome this barrier by capturing routinely entered outpatient data. Whilst the PowerForm's ability to increase EHR-based research output is exciting, its clinical audit function should not be overlooked. The audit exemplars we report - including reasons for outpatient referral, diagnostic categories and disposal decisions - appear mundane but this obscures the fact that information of this sort is almost never available to clinicians or hospital managers, particularly in settings where clinical documentation is paper-based or by free-text entry into computer systems. Understanding these patient profiles seems a basic requirement for effective organisation of outpatient services and provides a compelling rationale for further developing the EHR as a repository for routinely collected clinical data.

The clinical implications of our study are considerable because the technology applied in developing PowerForm and electronic transmission of the patient reports is potentially transportable and available for use in other non-cardiological outpatient settings. Wider development of PowerForms, or their equivalent in other hospital

For peer review only - http://bmjopen.bmj.com/site/about/guidelines.xhtml

systems, will build up the EHR and provide a substantial data resource for audit and research.

It was a limitation of this study that the GPs we surveyed were restricted to one clinical commissioning group in East London and the response rate of 44% leaves the results prone to response bias. As such, the generalisability of our findings will need testing in larger groups. The same can be said of our convenience sample of 53 patients approached at the end of their outpatient consultation. Whilst there were no refusals, it is important to be aware that these highly positive responses were obtained from a relatively small patient group. Additionally, whilst the results of this study did not show a significant difference in consultation times between PowerForm and paper-based consultation, comfort using computer systems is variable and the consultation times recorded in this study are not necessarily generalisable to all clinicians. A further limitation was the setting of the study in a general cardiology outpatient clinic and although it is likely that the technology is transportable to other clinical settings, this will need confirmation in future studies. Future studies of live use in other clinical settings will allow further evaluation of the added value offered by the PowerForm. Specifically, studies could be designed to ascertain the usefulness of the patient report as a communication tool between patients and physicians following the index encounter. Studies could also be designed to examine the utility of the patient reports as an educational resource for patients assessing whether patients had referred to them after their outpatient consultation.

For peer review only - http://bmjopen.bmj.com/site/about/guidelines.xhtml

CONCLUSIONS

Using a purpose built PowerForm, an automated patient report of a cardiac outpatient consultation can be developed providing a feasible alternative to the dictated clinic letter. This report is highly valued by both patients and GPs and is immediately available to both groups. The PowerForm encourages physicians to populate the EHR with coded data during real-time consultation. These data can then be de-identified, downloaded and used for audit and research. Implementation of the PowerForm across other specialties will allow healthcare professionals to capitalise on the benefits offered by the EHR.

LIST OF ABBREVIATIONS

EHR = Electronic Health Record, GP = General Practitioner, HIE = Health Information Exchange, EMIS = Egton Medical Information Systems, DTS = Data Transfer Service

DECLARATIONS

Ethics approval and consent to participate

Policy criterion: The work is primarily intended to improve local care, not provide generalizable knowledge in a field of inquiry.

Explanation: The work reported here meets this criterion because recording of outpatient data is a universally recommended practice. We sought only to evaluate improvements in recording of outpatient data using an electronic template and the advantages it presented in terms of communicating with primary care and providing data of potential value for audit and research.

For peer review only

For peer review only - http://bmjopen.bmj.com/site/about/guidelines.xhtml

Funding statement

This study was funded by The Guttmann Academic Partnership hosted by UCLPartners. NB had financial support from the Isaac Schapera Trust Fund for the submitted work. AML acknowledges support from the NIHR Cardiovascular Biomedical Research Unit at Barts Health NHS Trust and from the "SmartHeart" EPSRC programme grant (EP/P001009/1). AT and MRB acknowledge funding from the Medical Research Council (MR/K006584/1).

Data sharing statement

All data generated or analysed during this study are included in this published article [and its supplementary information files].

Competing interests

The authors declare that they have no competing interests.

Authors' contributions

NB designed data collection tools, monitored data collection for the whole study, wrote the statistical analysis plan, cleaned and analysed the data, and drafted and revised the paper. RAA designed data collection tools (creation of the PowerForm and utilisation over the period of the study) and cleaned and analysed the data. RW designed data collection tools (creation of the PowerForm and utilisation over the period of the study) and cleaned and analysed the data. MKDH designed data collection tools (surveys of feedback) and cleaned and analysed the data. MRB designed data collection tools and cleaned and analysed the data. AML designed data collection tools (download of data from PowerForms) and cleaned and analysed the data. SJ designed data collection tools (creation of PowerForm and SmartTemplate) and cleaned and analysed the data. CG designed data collection tools (creation of PowerForm and SmartTemplate) and cleaned and analysed the

For peer review only - http://bmjopen.bmj.com/site/about/guidelines.xhtml

data. JR designed data collection tools and cleaned and analysed the data. AT designed data collection tools, monitored data collection for the whole study, cleaned and analysed the data, and drafted and revised the paper. He is guarantor. All authors provided intellectual input into the draft of the manuscript, and read, edited and approved the final version.

Acknowledgements

MKDH was in part supported by the National Institute for Health Research (NIHR) Collaboration for Leadership in Applied Health Research and Care (CLAHRC) North Thames at Bart's Health NHS Trust. AT acknowledges support of Barts Cardiovascular Biomedical Research Unit, funded by the National Institute for Health Research.

For peer review only

REFERENCES

1. Gunter TD, Terry NP. The Emergence of National Electronic Health Record Architectures in the United States and Australia: Models, Costs, and Questions. J Med Internet Res. 7. Centre for Global eHealth Innovation, Toronto, Canada. 2005.

2. Yamamoto LG, Khan AN. Challenges of electronic medical record implementation in the emergency department. Pediatr Emerg Care. 2006;22(3):184-91; quiz 92.

3. Rawal J, Barnett P, Lloyd BW. Use of structured letters to improve communication between hospital doctors and general practitioners. Bmj. 1993;307(6911):1044.

4. Hook SE, Banister GC, Topliss C, Webb J. Letters and notes in orthopaedic surgery. Ann R Coll Surg Engl. 2006;88(3):292-6.

For peer review only - http://bmjopen.bmj.com/site/about/guidelines.xhtml

5. Parks T, Kingham E, McEwen D, Cooper S. The preference of general practitioners for structured outpatient clinic letters. Clin Med (Lond). 2011;11(2):205-6.

6. Peridis S, Roberts D, Hopkins C. Outpatient clinic letters in ENT. Is there any margin of improvement? B-ent. 2012;8(1):33-5.

7. The Electronic Health Records System In The UK. Centre for Public Impact. URL: https://www.centreforpublicimpact.org/case-study/electronic-health-records-system-uk. Accessed 30th November 2017.

8. Kruse CS, Kristof C, Jones B, Mitchell E, Martinez A. Barriers to Electronic Health Record Adoption: a Systematic Literature Review. J Med Syst. 2016;40(12):252.

9. Wells BJ, Chagin KM, Nowacki AS, Kattan MW. Strategies for handling missing data in electronic health record derived data. EGEMS (Wash DC). 2013;1(3):1035.

10. Sterne JA, White IR, Carlin JB, Spratt M, Royston P, Kenward MG, et al. Multiple imputation for missing data in epidemiological and clinical research: potential and pitfalls. Bmj. 2009;338:b2393.

11. NHS England. Five Year Forward View. NHS England; 2014. URL: www .england.nhs.uk/wp-content/uploads/2014/10/5yfv-web.pdf. Accessed 17th February 2017.

12. Blumenthal D, Tavenner M. The "meaningful use" regulation for electronic health records. N Engl J Med. 2010;363(6):501-4.

13. Buntin MB, Jain SH, Blumenthal D. Health information technology: laying the infrastructure for national health reform. Health Aff (Millwood). 2010;29(6):1214-9.

For peer review only - http://bmjopen.bmj.com/site/about/guidelines.xhtml

14. Centers for Medicare and Medicaid Services. Stage 2 Eligible Professional Meaning Use Core Measures Measure 8 of 17. 2012; 1–3.

15. Federman AD, Sanchez-Munoz A, Jandorf L, Salmon C, Wolf MS, Kannry J. Patient and clinician perspectives on the outpatient after-visit summary: a qualitative study to inform improvements in visit summary design. J Am Med Inform Assoc. 2016.

16. Dean SM, Gilmore-Bykovskyi A, Buchanan J, Ehlenfeldt B, Kind AJ. Design and Hospitalwide Implementation of a Standardized Discharge Summary in an Electronic Health Record. Jt Comm J Qual Patient Saf. 2016;42(12):555-AP11.

17. Carr LL, Zelarney P, Meadows S, Kern JA, Long MB, Kern E. Development of a Cancer Care Summary Through the Electronic Health Record. J Oncol Pract. 2016;12(2):e231-40.

18. Cerner Millenium. About Cerner. http://www.cerner.com/About_Cerner. Accessed 16th February 2017.

19. Cerner Millenium. Global Overview. http://www.cerner.com/About_Cerner/Corporate_Profile. Accessed 16th February 2017.

20. Revised Standards for Quality Improvement Reporting Excellence SQUIRE 2.0. http://www.squire-statement.org. Accessed 1st April 2017.

21. Collier, Ken W. (2011). Agile Analytics: A Value-Driven Approach to Business Intelligence and Data Warehousing. Pearson Education. pp. 121 ff. ISBN 9780321669544. What is a self-organizing team?

22. Data Transfer Service. IBM Bluemix. URL: https://knowledgelayer.softlayer.com/topic/data-transfer-service. Accessed 17th February 2017.

For peer review only - http://bmjopen.bmj.com/site/about/guidelines.xhtml

23. United Kingdom Health Departments' Research Ethics Service. URL: http://www.hra.nhs.uk/documents/2016/06/defining-research.pdf. Accessed 24th August 2017

24. Dean BB, Lam J, Natoli JL, Butler Q, Aguilar D, Nordyke RJ. Review: use of electronic medical records for health outcomes research: a literature review. Med Care Res Rev. 2009;66(6):611-38.

25. Cowie MR, Blomster JI, Curtis LH, Duclaux S, Ford I, Fritz F, et al. Electronic health records to facilitate clinical research. Clin Res Cardiol. 2017;106(1):1-9.

26. Cederholm S, Hill G, Asiimwe A, Bate A, Bhayat F, Persson Brobert G, et al. Structured assessment for prospective identification of safety signals in electronic medical records: evaluation in the health improvement network. Drug Saf. 2015;38(1):87-100.

27. Goldstein BA, Navar AM, Pencina MJ, Ioannidis JP. Opportunities and challenges in developing risk prediction models with electronic health records data: a systematic review. J Am Med Inform Assoc. 2017;24(1):198-208.

28. Denaxas SC, Morley KI. Big biomedical data and cardiovascular disease research: opportunities and challenges. Eur Heart J Qual Care Clin Outcomes 2015;1: 9-16.

For peer review only

Legends for illustrations

Figure 1. Sample patient report: Sides 1 and 2

Figure 2. GP questionnaire. Responses to "How would you rate the utility of this new Outpatient Report compared with the conventional typed letter posted to your practice?"

For peer review only - http://bmjopen.bmj.com/site/about/guidelines.xhtml

Figure 3. Aggregate analysis of data extracted from the PowerForm in 360 patients (A) Indications for outpatient referral (B) Diagnostic categories (C) Disposal decisions

ADDITIONAL MATERIAL

ADDITIONAL TABLE A1. Responses to GP questionnaires. File format: .doc

ADDITIONAL TABLE A2. Responses to patient questionnaires. File format: .doc

ADDITIONAL TABLE A3. Consultation times for patients seen in general cardiology clinics (A) Using the PowerForm and SmartTemplate (B) Using paper-based consultation and dictated clinic letter. File format: .doc

For peer review only

Side 1

Side 2

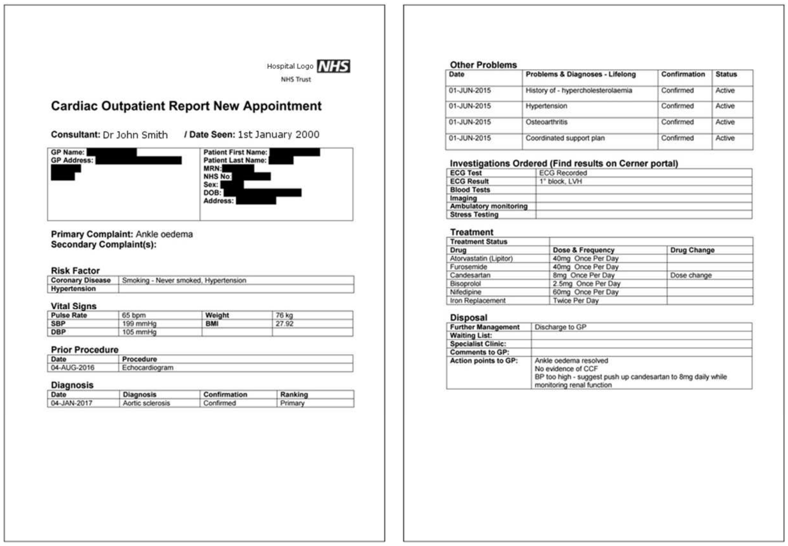

Hospital Logo NHS
NHS Trust

Cardiac Outpatient Report New Appointment

Consultant: Dr John Smith **/ Date Seen:** 1st January 2000

**GP Name:** [redacted]<br>**GP Address:** [redacted]	**Patient First Name:** [redacted]<br>**Patient Last Name:** [redacted]<br>**MRN:** [redacted]<br>**NHS No:** [redacted]<br>**Sex:** [redacted]<br>**DOB:** [redacted]<br>**Address:** [redacted]

Primary Complaint: Ankle oedema
Secondary Complaint(s):

Risk Factor

**Coronary Disease**	Smoking - Never smoked, Hypertension
**Hypertension**	

Vital Signs

**Pulse Rate**	65 bpm	**Weight**	76 kg
**SBP**	199 mmHg	**BMI**	27.92
**DBP**	105 mmHg		

Prior Procedure

Date	Procedure
04-AUG-2016	Echocardiogram

Diagnosis

Date	Diagnosis	Confirmation	Ranking
04-JAN-2017	Aortic sclerosis	Confirmed	Primary

Other Problems

Date	Problems & Diagnoses - Lifelong	Confirmation	Status
01-JUN-2015	History of - hypercholesterolaemia	Confirmed	Active
01-JUN-2015	Hypertension	Confirmed	Active
01-JUN-2015	Osteoarthritis	Confirmed	Active
01-JUN-2015	Coordinated support plan	Confirmed	Active

Investigations Ordered (Find results on Cerner portal)

**ECG Test**	ECG Recorded
**ECG Result**	1° block, LVH
**Blood Tests**	
**Imaging**	
**Ambulatory monitoring**	
**Stress Testing**	

Treatment

**Treatment Status**		
**Drug**	**Dose & Frequency**	**Drug Change**
Atorvastatin (Lipitor)	40mg Once Per Day	
Furosemide	40mg Once Per Day	
Candesartan	8mg Once Per Day	Dose change
Bisoprolol	2.5mg Once Per Day	
Nifedipine	60mg Once Per Day	
Iron Replacement	Twice Per Day	

Disposal

**Further Management**	Discharge to GP
**Waiting List:**	
**Specialist Clinic:**	
**Comments to GP:**	
**Action points to GP:**	Ankle oedema resolved<br>No evidence of CCF<br>BP too high - suggest push up candesartan to 8mg daily while monitoring renal function

Sample patient report: Sides 1 and 2

156x115mm (300 x 300 DPI)

ew only

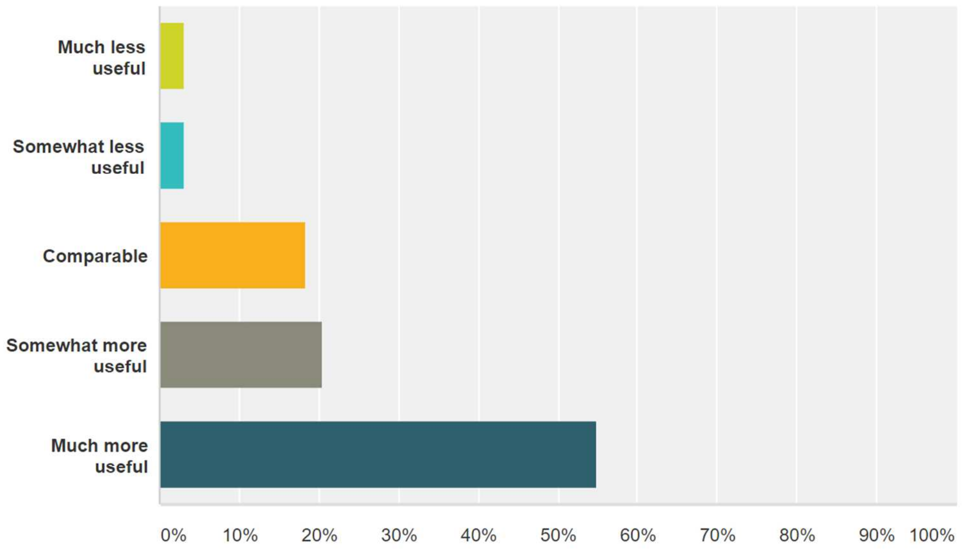

GP questionnaire. Responses to “How would you rate the utility of this new Outpatient Report compared with the conventional typed letter posted to your practice?”

111x65mm (300 x 300 DPI)

review only

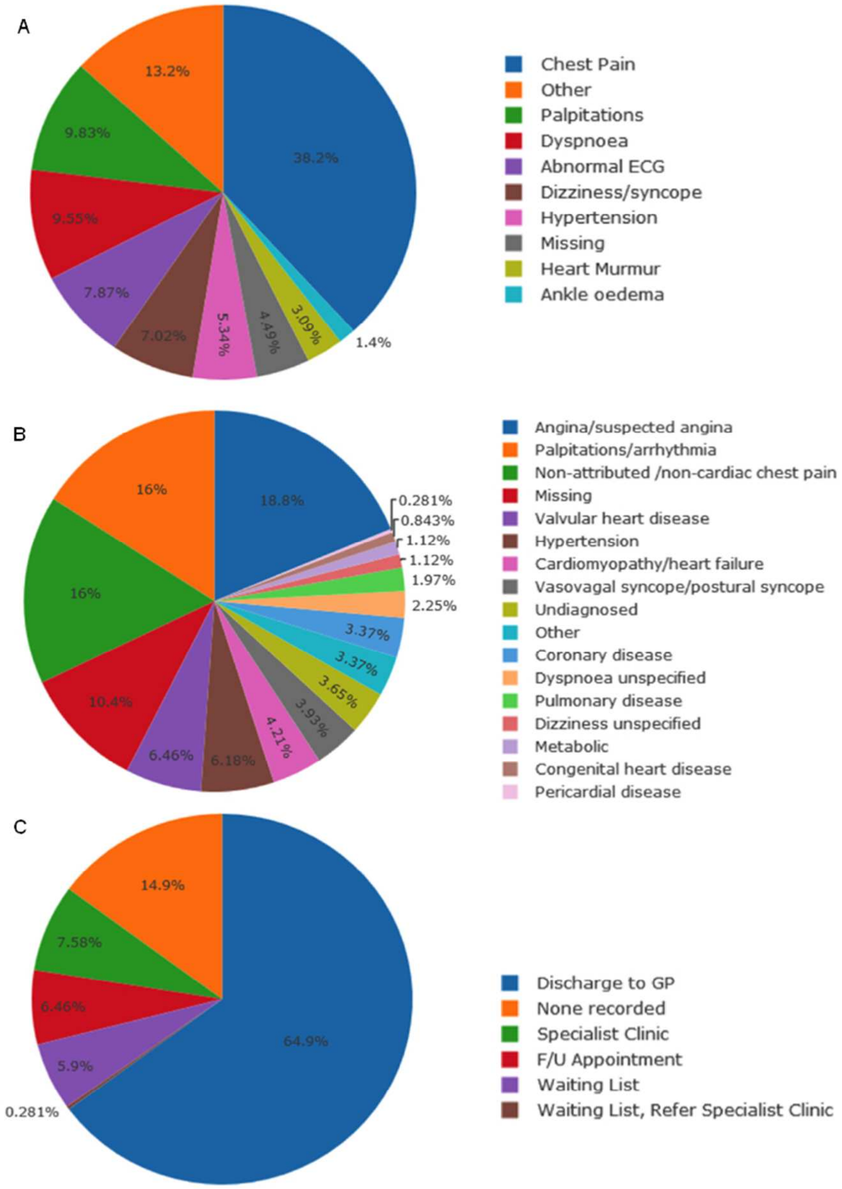

Aggregate analysis of data extracted from the PowerForm in 360 patients (A) Indications for outpatient referral (B) Diagnostic categories (C) Disposal decisions

71x91mm (300 x 300 DPI)

ADDITIONAL TABLE A1. Responses to GP questionnaires

Questions (posed to 93 GPs)	Cardiac outpatient report questionnaire for GPs				
How many GPs work in your practice? (93 responses)	**0-1**	**2-4**	**5+**		
	3	34	56		
	3.2%	36.6%	60.2%		
The patient is presented with a copy of the Outpatient Report on leaving the cardiac outpatient department. How important do you think this is? (93 responses)	**Not at all important**	**Slightly important**	**Somewhat important**	**Very important**	**Extremely important**
	0	0	15	48	30
	0.0%	0.0%	16.1%	51.6%	32.3%
The Outpatient Report is transmitted electronically to the patient's EMIS file immediately after the consultation. How important do you think same-day delivery of the Outpatient Report is? (89 responses)	**Not at all important**	**Slightly important**	**Somewhat important**	**Very important**	**Extremely important**
	0	4	10	34	41
	0.0%	4.5%	11.2%	38.2%	46.1%
The SNOMED codes used in the Outpatient Report will permit the transfer of data directly into the relevant EMIS fields. How useful do you think this will be? (92 responses)	**Not at all useful**	**Slightly useful**	**Somewhat useful**	**Very useful**	**Extremely useful**
	1	2	9	37	43
	1.1%	2.2%	9.8%	40.2%	46.7%
Is the layout of the Outpatient Report easy to follow? (89 responses)	**Yes**	**No**	**Somewhat**		
	74	2	13		
	83.1%	2.2%	14.6%		
Does the content of the Outpatient Report provide adequate information for your clinical needs? (92 responses)	**Yes**	**No**	**Somewhat**		
	69	5	18		
	75.0%	5.4%	19.6%		
The Outpatient Report provides a list of investigations and medications that a patient will receive after their clinic appointment. How useful do you think these sections are for your further follow up of patients? (88 responses)	**Not at all useful**	**Slightly useful**	**Somewhat useful**	**Very useful**	**Extremely useful**
	0	0	6	35	47
	0.0%	0.0%	6.8%	39.8%	53.4%
How would you rate the utility of this new Outpatient Report compared with the conventional typed letter posted to your practice? (93 responses)	**Much less useful**	**Somewhat less useful**	**Comparable**	**Somewhat more useful**	**Much more useful**
	3	3	17	19	51
	3.2%	3.2%	18.3%	20.4%	54.8%

For peer review only

ADDITIONAL TABLE A2. Responses to patient questionnaires

Questions (posed to 53 Patients)	Cardiac outpatient report questionnaire for patients				
Which age group are you a part of? (53 responses)	**16-35**	**36-55**	**56-75**	**76+**	**Prefer not to say**
	7	14	24	8	0
	13.2%	26.4%	45.3%	15.1%	0.0%
What is your gender? (53 responses)	**Male**	**Female**	**Prefer not to say**		
	26	27	0		
	49.1%	50.9%	0.0%		
As a patient, you are given a copy of the Outpatient Report on leaving the cardiac outpatient department. How important do you think this is? (53 responses)	**Not at all important**	**Slightly important**	**Somewhat important**	**Very important**	**Extremely important**
	1	0	3	29	20
	1.9%	0.0%	5.7%	54.7%	37.7%
Is the layout of the Outpatient Report easy to follow? (53 responses)	**Yes**	**No**	**Somewhat**		
	52	0	1		
	98.1%	0.0%	1.9%		
How would you rate this new Outpatient Report compared with the conventional typed letter posted to your home? (53 responses)	**Much less useful**	**Somewhat less useful**	**Comparable**	**Somewhat more useful**	**Much more useful**
	0	0	4	12	37
	0.0%	0.0%	7.5%	22.6%	69.8%
Do you think this Outpatient Report will help you to understand your medical condition? (53 responses)	**Yes**	**No**	**Somewhat**		
	49	1	3		
	92.5%	1.9%	5.7%		
Does the Outpatient Report help you understand the follow up you will receive after your consultation? (53 responses)	**Yes**	**No**	**Somewhat**		
	49	3	1		
	92.5%	5.7%	1.9%		

For peer review only

ADDITIONAL TABLE A3. Consultation times for patients seen in general cardiology clinics (A) Using the PowerForm and SmartTemplate (B) Using paper-based consultation and dictated clinic letter.

(A) SmartTemplate		(B) Dictated Clinic Letter	
Patient	Consultation Time (minutes: seconds)	Patient	Consultation Time (minutes: seconds)
1	11:03	1	14:07
2	15:03	2	18:48
3	23:26	3	15:57
4	14:41	4	11:33
5	10:22	5	12:57
6	15:21	6	13:59
7	14:36	7	19:14
8	11:28	8	15:27
9	14:41	9	11:12
10	16:53	10	10:48
11	11:21	11	10:11
12	12:02	12	12:38
13	08:29	13	13:36
14	12:28	14	13:26
15	20:01	15	12:41
16	14:16	16	21:13
17	12:34	17	12:05
18	12:35	18	16:27
19	11:36	19	10:53
20	19:27	20	14:09
21	11:26	21	13:50
22	13:34	22	17:43

For peer review only

**Mean consultation time**		13:58		14:13
**Standard deviation**		03:30		02:57

For peer review only

Text section and item name	Section or item description	Included?		Page number
Notes to authors	‣ The SQUIRE guidelines provide a framework for reporting new knowledge about how to improve healthcare.			
	‣ The SQUIRE guidelines are intended for reports that describe system level work to improve the quality, safety and value of healthcare, and used methods to establish that observed outcomes were due to the intervention(s).			
	‣ A range of approaches exists for improving healthcare. SQUIRE may be adapted for reporting any of these.			
	‣ Authors should consider every SQUIRE item, but it may be inappropriate or unnecessary to include every SQUIRE element in a particular manuscript.			
	‣ The SQUIRE glossary contains definitions of many of the key words in SQUIRE.			
	‣ The explanation and elaboration document provides specific examples of well-written SQUIRE items and an in-depth explanation of each item.			
	‣ Please cite SQUIRE when it is used to write a manuscript.			
Title and abstract				
1. Title	Indicate that the manuscript concerns an initiative to improve healthcare (broadly defined to include the quality, safety, effectiveness, patient-centredness, timeliness, cost, efficiency and equity of healthcare).	Yes	Discussed that we are enhancing communication to improve healthcare	0
2. Abstract	a. Provide adequate information to aid in searching and indexing.	Yes		1

For peer review only - http://bmjopen.bmj.com/site/about/guidelines.xhtml

	b. Summarise all key information from various sections of the text using the abstract format of the intended publication or a structured summary such as: background, local problem, methods, interventions, results, conclusions.	Yes		1
Introduction	*Why did you start?*			
3. Problem description	Nature and significance of the local problem.	Yes	EHR under-utilised within the NHS. OP consultations don't contribute. Communication difficulties with primary care & patient.	3
4. Available knowledge	Summary of what is currently known about the problem, including relevant previous studies.	Yes	After visit summary used in USA. Citations highlighting the use of the EHR for inpatient discharge summaries and cancer clinics. No studies in cardiology.	4
5. Rationale	Informal or formal frameworks, models, concepts and/or theories used to explain the problem, any reasons or assumptions that were used to develop the intervention(s) and reasons why the intervention(s) was expected to work	Yes	SNOMED-based electronic PowerForm comprising a	4

			user-friendly interface for real-time entry of clinical data into Millennium during consultation in a general cardiac outpatient clinic	
6. Specific aims	Purpose of the project and of this report.	Yes		4
Methods	*What did you do?*			
7. Context	Contextual elements considered important at the outset of introducing the intervention(s).	Yes	Description of Cerner. Also discussed that this was used in general cardiology clinics at Barts Health NHS Trust	4-6
8. Intervention(s)	a. Description of the intervention(s) in sufficient detail that others could reproduce it.	Yes	Development of PowerForm, smart template, transfer + data download methodology	5-8
	b. Specifics of the team involved in the work.	Yes	3 cardiologists	6

For peer review only - http://bmjopen.bmj.com/site/about/guidelines.xhtml

9. Study of the intervention(s)	a. Approach chosen for assessing the impact of the intervention(s).	Yes	Electronic transfer, data download	4+5
	b. Approach used to establish whether the observed outcomes were due to the intervention(s).	Yes	Data download, electronic transfer, satisfaction surveys, consultation times	5-8
10. Measures	a. Measures chosen for studying processes and outcomes of the intervention(s), including rationale for choosing them, their operational definitions and their validity and reliability.	Yes	Surveys of feedback, download of data, electronic transfer, consultation times	5-8
	b. Description of the approach to the ongoing assessment of contextual elements that contributed to the success, failure, efficiency and cost.	Yes		
	c. Methods employed for assessing completeness and accuracy of data.	Yes		
11. Analysis	a. Qualitative and quantitative methods used to draw inferences from the data.	Yes	Ordinal logistic regression, mean consultation times ± SD	7-8
	b. Methods for understanding variation within the data, including the effects of time as a variable.	Yes	Used ordinal logistic regression to	9

			determine relationship between patient demographics & utility of report versus dictated letter	
12. Ethical considerations	Ethical aspects of implementing and studying the intervention(s) and how they were addressed, including, but not limited to, formal ethics review and potential conflict(s) of interest.	Yes	Ethics statement, patient/GP surveys anonymised & voluntary	7-8
Results	*What did you find?*			
13. Results	a. Initial steps of the intervention(s) and their evolution over time (eg, time-line diagram, flow chart or table), including modifications made to the intervention during the project.	Yes	Usage of the PowerForm over an 8 month time period	9
	b. Details of the process measures and outcomes.	Yes	Usage of PowerForm, data download, electronic transfer, consultation times, feedback	9-11
	c. Contextual elements that interacted with the intervention(s).	Yes	Ordinal logistic regression for	10

			patient age/gender & thought's on utility of OP report	
	d. Observed associations between outcomes, interventions and relevant contextual elements.	Yes	No association between patient demographics & thoughts on utility of OP report	10
	e. Unintended consequences such as unexpected benefits, problems, failures or costs associated with the intervention(s).	Yes	Effect of PowerForm use on consultation times. Also examined electronic transfer capability.	9
	f. Details about missing data.	Yes	Addressed that we had a low response rate from GPs and that patient sample size was small	14
Discussion	*What does it mean?*			

For peer review only - http://bmjopen.bmj.com/site/about/guidelines.xhtml

14. Summary	a. Key findings, including relevance to the rationale and specific aims.	Yes	First paragraph of discussion	11
	b. Particular strengths of the project.	Yes	Helps the NHS become paperless, data download fully successful and patient/GP satisfaction very high	11-12
15. Interpretation	a. Nature of the association between the intervention(s) and the outcomes.	Yes	Intervention enabled data download and electronic transfer. Intervention was also highly rated by GPs & patients. No significant difference in consultation times.	11-13
	b. Comparison of results with findings from other publications.	Yes	Discussed how our findings can be used to improve the repository of data within the EHR. Also	11-13

			discussed reasons why the PowerForm utilisation rate was initially low but gradually increased over the course of the study.	
	c. Impact of the project on people and systems.	Yes	Use in audit/research	13
	d. Reasons for any differences between observed and anticipated outcomes, including the influence of context.	Yes	Consultant uptake increased over the 8 month time period, perhaps reflecting increased familiarity with the software	12
	e. Costs and strategic trade-offs, including opportunity costs.	Yes	Despite the learning curve associated with its use, the PowerForm may cut overall time taken to dictate a clinic letter	12

16. Limitations	a. Limits to the generalisability of the work.	Yes	Low response rate, cardiac outpatient setting, discussed the need for future studies of live use	14
	b. Factors that might have limited internal validity such as confounding, bias or imprecision in the design, methods, measurement or analysis.	Yes	Discussed that patient sample was a convenience sample. Also discussed that our consultation time findings were found with a cardiologist who was familiar with the PowerForm	14
	c. Efforts made to minimise and adjust for limitations.	Yes	PowerForms were available to 3 participating cardiologists	6

17. Conclusions	a. Usefulness of the work.	Yes	Discussed that the PowerForm enables data download, direct electronic transfer. Also discussed that patient report is highly valued by patients and GPs.	15
	b. Sustainability.	Yes	Described how the PowerForm is a feasible alternative to the dictated clinic letter. Also described that we used it over an 8 month time period	15
	c. Potential for spread to other contexts.	Yes	Other specialties	13+15
	d. Implications for practice and for further study in the field.	Yes	Low GP response rate mentioned as further studies in this group.	14

			Also implications for audit/research & communication	
	e. Suggested next steps.	Yes	Discussion about how integration will allow us to benefit from EHR	15
Other information				
18. Funding	Sources of funding that supported this work. Role, if any, of the funding organisation in the design, implementation, interpretation and reporting.	Yes	Mentioned during online submission process & funding statement	16

For peer review only - http://bmjopen.bmj.com/site/about/guidelines.xhtml
